# Exploring the Effects of a Social Robot's Speech Entrainment and Backstory on Young Children's Emotion, Rapport, Relationship, and Learning

**DOI:** 10.3389/frobt.2019.00054

**Published:** 2019-07-09

**Authors:** Jacqueline M. Kory-Westlund, Cynthia Breazeal

**Affiliations:** MIT Media Lab, Massachusetts Institute of Technology, Cambridge, MA, United States

**Keywords:** children, entrainment, language development, peer modeling, rapport, relationship, robotics, storytelling

## Abstract

In positive human-human relationships, people frequently mirror or mimic each other's behavior. This mimicry, also called entrainment, is associated with rapport and smoother social interaction. Because rapport in learning scenarios has been shown to lead to improved learning outcomes, we examined whether enabling a social robotic learning companion to perform rapport-building behaviors could improve children's learning and engagement during a storytelling activity. We enabled the social robot to perform two specific rapport and relationship-building behaviors: speech entrainment and self-disclosure (shared personal information in the form of a backstory about the robot's poor speech and hearing abilities). We recruited 86 children aged 3–8 years to interact with the robot in a 2 × 2 between-subjects experimental study testing the effects of robot entrainment *Entrainment* vs. *No entrainment* and backstory about abilities *Backstory* vs. *No Backstory*. The robot engaged the children one-on-one in conversation, told a story embedded with key vocabulary words, and asked children to retell the story. We measured children's recall of the key words and their emotions during the interaction, examined their story retellings, and asked children questions about their relationship with the robot. We found that the robot's entrainment led children to show more positive emotions and fewer negative emotions. Children who heard the robot's backstory were more likely to accept the robot's poor hearing abilities. Entrainment paired with backstory led children to use more of the key words and match more of the robot's phrases in their story retells. Furthermore, these children were more likely to consider the robot more human-like and were more likely to comply with one of the robot's requests. These results suggest that the robot's speech entrainment and backstory increased children's engagement and enjoyment in the interaction, improved their perception of the relationship, and contributed to children's success at retelling the story.

## 1. Introduction

Social robots have been designed as peers, tutors, and teachers to help children learn a variety of subjects (Belpaeme et al., 2018), including math (Clabaugh et al., 2015; Kennedy et al., 2015), language (Movellan et al., 2009; Kory and Breazeal, 2014; Gordon et al., 2016; Kory Westlund et al., 2017a,b; Vogt et al., 2017; Rintjema et al., 2018), reading (Gordon and Breazeal, 2015), handwriting (Hood et al., 2015), social skills (Robins et al., 2005; Scassellati et al., 2018), curiosity (Gordon et al., 2015), and a growth mindset (Park et al., 2017b). Prior work has explored how social robots can best engage children in learning activities and improve learning outcomes, using, e.g., personalization of behavior or curriculum (Gordon and Breazeal, 2015; Hood et al., 2015; Gordon et al., 2016; Baxter et al., 2017; Scassellati et al., 2018), appealing appearance and personality (Kory and Breazeal, 2014), and appropriate nonverbal behaviors (Kennedy et al., 2015; Kory Westlund et al., 2017a,b). One aspect of human-human interpersonal interaction that has been linked to improved learning outcomes in peer tutoring situations is rapport and positive relationships (Sinha and Cassell, 2015a,b). Because of this link, we hypothesize that improving a social robot's capabilities for building rapport and positive relationships with children may similarly lead to improved learning outcomes.

Some prior work with adults provides evidence in support of this hypothesis (Kidd and Breazeal, 2008; Lubold et al., 2016, 2018; Lubold, 2017); however, there is little work yet exploring a social robot's rapport and relationship with young children. Thus, in this paper, we explored whether enabling a social robot to perform rapport-building behaviors, including speech and behavior entrainment, and giving the robot an appropriate backstory regarding its abilities, could help establish rapport and generate positive interactions with children, which we hypothesized could improve children's learning and engagement.

## 2. Background

### 2.1. Relationships, Rapport, and Learning

We have strong evidence that children's peer relationships provide bountiful opportunities for learning via observing peers, being in conflict with peers, and cooperating with peers (Piaget, 1932; Bandura and Walters, 1963; Bandura, 1971; Vygotsky, 1978; Tudge and Rogoff, 1989; Rubin et al., 1998; De Lisi and Golbeck, 1999; Whitebread et al., 2007). The research so far on children's peer learning discusses how children might learn from other, but does not yet thoroughly address what precisely modulates peer learning. That is: Are all peers approximately equivalent as sources to promote learning, or is there something about some peers that makes them “better inputs” than others? In the context of social robots, what is it about a social robot that could lead children to learn more, or less?

Two possible modulating factors are rapport and a positive relationship. Some recent work has linked rapport to improved learning outcomes in older children's human-human peer tutoring situations (Sinha and Cassell, 2015a,b). In addition, the social bonds between children and teachers can predict learner performance (Wentzel, 1997). Other research has shown that children may learn math concepts from media characters more effectively when they have stronger parasocial relationships with those characters (Gola et al., 2013; Richards and Calvert, 2017).

Many different social and relational factors can increase rapport, trust, and engagement with virtual agents and robots. For example, using appropriate social cues (Desteno et al., 2012; Lee et al., 2013; Breazeal et al., 2016b), contingent backchanneling (Park et al., 2017a), nonverbal mirroring (Bailenson et al., 2005; Burleson and Picard, 2007; Lubold et al., 2018), responsiveness and proactivity (Kim et al., 2006), increased social presence (Lester et al., 1997), and matching ethnic communication styles (Cassell et al., 2009) all have had positive effects.

We chose to implement two rapport- and relationship-building behaviors in a social robot to explore their effects on young children's engagement and learning: speech entrainment and self-disclosure (shared personal information).

### 2.2. Speech Entrainment

In positive human-human interpersonal interactions, people frequently mimic each other's behavior—such as posture, affect, speech patterns, gestures, facial expressions, and more—unconsciously, without awareness or intent (Davis, 1982; Grammer et al., 1998; Philippot et al., 1999; Provine, 2001; Lakin et al., 2003; Semin and Cacioppo, 2008; Reitter et al., 2011; Borrie and Liss, 2014). This mimicry, also called entrainment, is considered a signal of rapport and has been observed in a variety of human relationships (Tickle-Degnen and Rosenthal, 1990; Dijksterhuis and Bargh, 2001; Rotenberg et al., 2003; Dijksterhuis, 2005; Chartrand and van Baaren, 2009; Wiltermuth and Heath, 2009; Lubold, 2017), as well as with robots and virtual agents (Breazeal, 2002; Bell et al., 2003; Suzuki and Katagiri, 2007; Levitan et al., 2016). While there is less work exploring mimicry and rapport in children, there is some showing that infants and children mimic emotions with humans (Haviland and Lelwica, 1987; Chisholm and Strayer, 1995; Rotenberg et al., 2003) and with robots (Gordon et al., 2016). Thus, enabling a robot to perform entrainment could significantly increase children's rapport with it. We chose speech entrainment because language learning is often a dialogue-heavy activity, and thus, would perhaps be more noticeable and relevant than entraining other behaviors. In addition, given the morphology and technical limitations of the robot platform we had available for this study (the Tega robot, described below), speech entrainment was one of the most feasible behaviors to study, though other behaviors could also be examined in the future (such as posture or affect).

Speech entrainment involves matching the vocal features such as speaking rate, intensity, pitch, volume, and prosody of one's interlocutor. This mimicry tends to happen unconsciously, and more often when rapport has been established—i.e., when one feels closer to or more positively about one's interlocutor (Porzel et al., 2006; Reitter et al., 2011; Borrie and Liss, 2014). Some recent work has explored increasing prosodic synchrony in a speech-controlled child-robot game in order to promote cooperation and improve enjoyment (Chaspari and Lehman, 2016; Sadoughi et al., 2017). In addition, Lubold and colleagues developed several social voice-adaptive robots that adjust the pitch of the robot's text-to-speech voice to match that of its human interlocutor (Lubold et al., 2015, 2016, 2018; Lubold, 2017). This vocal entrainment contributed to increased learning with undergraduate students as well as middle school students during math tasks, but did not increase self-reported rapport. However, our work differs in several ways. We are investigating the impact of entrainment with younger children in a more social task—language learning—that may be more affected by social relationships. Second, these prior studies compared a robot with a text-to-speech voice to one that had a more expressive (albeit contingently adapted) voice. They did not control for the expressivity of the voice. Other recent work found that a robot with a more expressive voice was more effective as a learning companion, leading to greater engagement and learning, than a robot that used a flat voice, similar to a classic text-to-speech voice (Kory Westlund et al., 2017b). This work raises the question of whether the effects seen in Lubold et al.'s studies are strictly a result of the entrainment or a result of the robot's voice being more expressive. In the work presented here, we control for the robot's expressivity.

### 2.3. Backstory (Personal Self-Disclosure)

Backstory is the story told by or about an agent, including personal story (e.g., origin, family, hobbies), capabilities, limitations, and any other personal information that might be disclosed. With young children in particular, we expect that sharing information about an agent in a story context could make it easier for children to understand.

Prior work has shown that the story told about a robot prior to interaction can change how people perceive the robot and interact with it. Telling participants that a robot is a machine vs. a human-like, animate agent (Stenzel et al., 2012; Klapper et al., 2014; Kory Westlund et al., 2016b) or giving the robot a name and a story involving greater agency and experience (Darling et al., 2015) can manipulate people's perceptions of the robot as an animate, social agent as well as their empathy for the agent. These studies build on extensive work in social cognition and social psychology literature regarding the idea that framing or priming can influence subsequent behavior and perception (Dijksterhuis and Bargh, 2001; Biernat, 2004). However, it is not only stories told before an interaction, but also the content *of* an interaction that affects people's perceptions of their interlocutor. For example, one aspect of children's friendships and positive relationships is self-disclosure. Children disclose more information, and more personal information, in closer relationships (Rotenberg and Mann, 1986; Rotenberg, 1995). The amount of disclosure during conversation reflects how close two children feel to one another. A robot that discloses personal information may impact not only relationship formation and perception, but the story it tells could also impact how a child perceives how social an agent the robot is.

Backstory can also increase engagement with an agent. For example, in one study, giving a robot receptionist a scripted backstory during a long-term deployment increased engagement, since the story added interesting variation and history to the interactions people had with it (Gockley et al., 2005). However, no research as yet has examined the impact a backstory can have on young children's learning.

Part of our goal in giving the robot a backstory was to promote a more positive relationship. Thus, we examined specific interventions regarding the acceptance of peers and how these interventions might play into the story told about the robot. Favazza and colleagues explored how to promote the acceptance of peers with disabilities in children's kindergarten classrooms, as well as how to measure that acceptance (Favazza and Odom, 1996; Favazza et al., 2000). One component of the intervention they used involved telling stories with guided discussion about children with disabilities; a second component involved structured play with the peers who had disabilities. We combined the idea of telling a story about one of the robot's relevant difficulties that could be perceived as a disability—namely, its hearing and listening abilities—with the idea of self-disclosure as a component of children's friendships; and followed this story/disclosure with several structured activities with the robot.

There are ethical concerns regarding deception when giving robots stories that may elicit empathy, trust, or acceptance. In this study, the backstory we chose to use was fairly reflective of the actual limitations and capabilities of social robots. It pertained to the robot's difficulties with hearing and listening and was thus fairly realistic and not particularly deceptive, given general difficulties in social robotics with automatic speech recognition and natural language understanding. The remainder of the backstory discussed the robot's interest in storytelling and conversation, which was deceptive in that robots do not really have interests, but served to present the robot as a character with interests in these subjects in order to promote engagement in learning activities.

## 3. Methodology

### 3.1. Research Questions

We wanted to explore whether a social robot that entrained its speech and behavior to individual children and provided an appropriate backstory about its abilities could increase children's rapport, positive relationship, acceptance, engagement, and learning with the robot during a single session.

### 3.2. Design

The experiment included two between-subjects conditions: Robot entrainment (*Entrainment* vs. *No entrainment*) and Backstory about abilities (*Backstory* vs. *No Backstory*). We abbreviate the four conditions as *E-B, E-NB, NE-B*, and *NE-NB*. In the *Entrainment (E)* condition, the robot's speech was entrained based on each child's speaking rate, pitch, and volume, and exuberance. In the *Backstory (B)* condition, the experimenter explained that the robot was not so good at hearing and needed practice; this backstory was reinforced by the robot later.

### 3.3. Participants

We recruited 95 children aged 3–8 years (47 female, 48 male) from the general Boston area to participate in the study. We recruited a wide age range in order to recruit a sufficient number of participants and also because we were interested in seeing whether older children (e.g., 6–8 years) or younger children (e.g., 3–5 years) might relate differently to the robot's relational behavior, since children may develop relationships differently as they grow older (Hartup et al., 1988; Rubin et al., 1998).

Nine children were removed from analysis because they did not complete the study^1^. The children in the final sample included 86 children aged 3–8 (44 female, 42 male), with a mean age of 5.31 years (*SD* = 1.43). Of these, 3 were 3-year-olds, 30 were 4-year-olds, 19 were 5-year-olds, 15 were 6-year-olds, and 9 were 7-year-olds, and 10 were 8-year-olds. Forty-nine children spoke English only; 37 children were bilingual.

We used random counterbalanced assignment to assign children to conditions. There were 20 in the *E-B* condition, 16 in the *E-NB* condition; 28 children in the *NE-B* condition; and 22 in the *NE-NB* condition. The imbalance was a result of the children who did not complete the study. Table 1 lists age, gender, and bilingual status by condition. Age did not significantly differ by condition. We asked parents to rate their children's social behavior on a variety of dimensions; these ratings also did not significantly differ by condition.

**Table 1:** Demographic information about the participants by condition.

**Condition**	**Mean age (SD)**	**Girls**	**Boys**	**Monolingual**	**Bilingual**
E-B	5.40 (1.54)	11	9	12	8
E-NB	5.21 (1.34)	7	9	9	7
NE-B	5.44 (1.67)	13	15	18	10
NE-NB	5.27 (1.35)	13	9	11	11

Children's parents gave written informed consent prior to the start of the study, and all children assented to participate. The protocol was approved by the MIT Committee on the Use of Humans as Experimental Subjects.

### 3.4. Hypotheses

We expected that the robot's entrainment and backstory might affect both children's rapport and social behavior, as well as learning and retention, during a single session with the robot. Accordingly, we used a variety of measures to explore the effects of the robot's entrainment and backstory. We tentatively expected the following results:

**Learning**

- **H1:** In all conditions, children would learn the target vocabulary words presented in the robot's story. In prior studies, we have seen children learn new words from stories told by robots (Kory, 2014; Kory Westlund et al., 2017b; Park et al., 2019). However, we expected that children would learn more as a result of the robot's entrainment or from an increased relationship, i.e., the most in the *E-B* condition, followed by the *E-NB* and *NE-B* conditions, and the least in the *NE-NB* condition.
- **H2:** Children who learned the target vocabulary words would also use them in their story retells. We have previously seen children mirror a robot's vocabulary words in their own stories (Brennan, 1996; Iio et al., 2015; Kory Westlund et al., 2017b).
- **H3:** Because of the expected connection between the robot's entrainment and backstory to children's rapport and relationship, as well as prior work showing that the story told about a computer's limitations influenced participants' lexical entrainment (Pearson et al., 2006), we expected the entrainment and backstory would lead to differences in children's mirroring of the robot's story in their retells. Children in the *E-B* condition would produce more vocabulary, longer stories, and phrase mirroring because of more rapport and a closer relationship.

**Rapport, Relationship, and Social Behavior**

- **H4:** A robot with an appropriate backstory about its abilities (*E-B* and *NE-B* conditions) would lead to greater acceptance by children of the robot and more helping behaviors.
- **H5:** Both entrainment and backstory would lead children to treat the robot as a greater social other, such as laughing and smiling more (Provine, 2001; Smidl, 2006), and affording the robot courtesies such as saying goodbye or considering its preferences (Reeves and Nass, 1996). We expected to see this more in the *E-B* than the other conditions; and least in the *NE-NB* condition.
- **H6:** Children would show greater rapport, entrainment, mirroring, and helping behaviors with a robot that entrained to them (*E-B* and *E-NB* conditions). We also expected that a robot with both an appropriate backstory and entrainment (*E-B*) would promote a stronger relationship, and as a result, greater attention, engagement, rapport, and mirroring than in the *E-NB* condition. Furthermore, children's attention, engagement, and positive emotions would increase—or at least decrease less—over the course of the session than in the other conditions.
- **H7:** Children who reported a closer relationship to the robot would also show more mirroring behaviors, more helping behaviors, greater rapport, greater engagement, and more learning. We expected a connection between children's relationship and their learning because of prior work showing that rapport can facilitate learning in peer tutoring scenarios (Sinha and Cassell, 2015a,b).

### 3.5. Procedure

**Figure 1:**
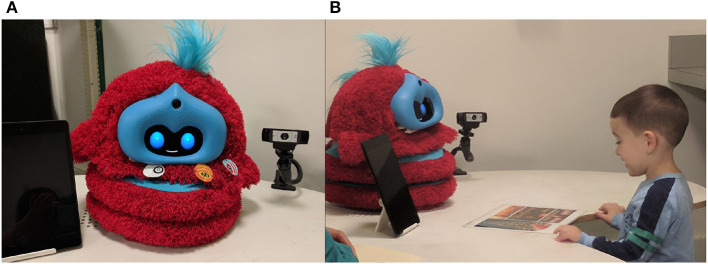
**(A)** The robot was placed on a table. The tablet was set upright to the left (when facing the robot), and the camera behind the robot and to the right. **(B)** A child discusses holidays with the robot in the picture conversation task. Written informed consent was obtained to use this image.

Five different experimenters (three female adults and two male adults) ran the study in pairs in a quiet room in the lab. The study setup is shown in Figure 1. One experimenter interacted with the child. The second experimenter was present in the room, but sat back behind a laptop and did not interact directly with the child; their role was to teleoperate the robot and manage the other equipment. Some children wished their parents to stay with them (e.g., if they were particularly shy); in these cases children's parents were instructed to watch only and let their children do as much as possible by themselves.

For each child, the interaction with the robot lasted about 20 min, followed by 5–10 min for the posttests. The interaction script, full interaction procedure, and other study materials are available for download from figshare at: https://doi.org/10.6084/m9.figshare.7175273; they are available for download as Supplementary Materials.

The experimenter introduced the sleeping robot, Tega, to the child and explained that it liked looking at pictures and telling stories. If the child was in the Backstory condition, the experimenter also explained that Tega sometimes had trouble hearing: “Do you see Tega's ears? Tega's ears are hiding under all the fur, so sometimes Tega's ears don't work very well. Tega sometimes has a lot of trouble hearing. You should talk to Tega in a loud and clear voice so Tega can hear you. Try to be understanding if Tega needs to hear something again.” Then, in all conditions, the experimenter invited the child to help wake up the robot.

The robot interaction had four main sections: A brief introductory conversation (providing context for sharing the backstory, 2–3 min), a conversation about pictures (providing opportunities for speech entrainment and a helping/compliance request, 5–6 min), a sticker task (a sharing/compliance request, 1 min), a storytelling activity (providing opportunities to learn words and mirror the robot's speech, 10–12 min), and a brief closing conversation (1–2 min).

In the introductory conversation, the robot introduced itself, shared personal information about its favorite color and an activity it liked doing, and prompted the child for disclosure in return. Then, in the Backstory condition, the robot reinforced the backstory provided by the experimenter earlier, telling the child, “Sometimes I have trouble hearing and I can't always understand what people tell me. I try really hard, but sometimes I just don't hear things right. I need help and practice to get better!”

The picture conversation took approximately 5 min and was designed to provide many conversation turns for the child, and thus provide the robot with opportunities to entrain its speech to the child's. The experimenter placed photos one at a time in front of the robot and child (e.g., a collage of holidays or pictures from children's movies). For each picture, the robot introduced the picture content, expressed something it liked about the picture, asked the child a question, responded with generic listening responses (e.g., “Can you tell me more?,” “Oh, cool!,” “Keep going!”), shared another fact relevant to the picture, and asked another question. At two points during this activity, there were scripted moments where the robot had difficulty hearing (saying, e.g., “I didn't hear that, can you say it again?”), to reinforce its backstory. The experimenter explained that the robot and child had to do at least three pictures, but they could do one more if they wanted—this set up a later compliance/helping task after the third picture, in which the robot asked if the child would do a fourth picture with it to help it practice extra. If the child declined the fourth picture, the experimenter moved on.

The sticker task was used to see how likely the child was to agree to a request by the robot to share a favorite object. The child was allowed to pick out a sticker from a small selection. The robot stated that it wanted the child's sticker and asked for it. The child could spontaneously speak or give their sticker to the robot, or decline. If the child gave their sticker, the experimenter would conveniently find a duplicate sticker in their pocket to replace it, so that the child would not have to forgo their favorite sticker.

The storytelling activity was modeled after the story retelling task used in Kory Westlund et al. (2017b). The robot told a story consisting of a 22-page subset of the wordless picture book “Frog, Where Are you?” by Mercer Mayer. The pages of the book were shown one at a time on the tablet screen. On each page, the robot said 1–2 sentences of the story. Every few pages, the robot asked a dialogic reading comprehension question about the events in the story, e.g., “Where is the deer taking the boy?,” 'and “How do you think the boy feels now?” (3 questions total, decreased from the 11 questions in the prior study to decrease the length of the story activity). As in the prior study, the robot responded to children's answers with encouraging, non-committal phrases such as “Mmhm,” “Good thought,” and “You may be right.”

We embedded six target vocabulary words (all nouns) into the story. As in the prior study, we did not test children on their knowledge of these words prior to the storytelling activity because we did not want to prime children to pay attention to these words, since that could bias our results regarding whether or not children would learn or use the words after hearing them in the context of the robot's story. We used the six key nouns identified in the original story in Kory Westlund et al. (2017b), which were replaced with the target words “gopher”(original word: animal), “crag” (rock),“lilypad” (log), “hollow” (hole), “antlers” (deer), and “cliff” (hill).

After the robot told the story, the robot prompted children to retell the story. Children could use the tablet while retelling the story to go through the story pages, so they could see the pictures to help them remember the story. Twice during the retell, the robot had difficulty hearing (“What? Can you say that again?”), which reinforced the backstory. Children's retellings were used as a measure of their story recall, mirroring of the robot's speech, and expressive use of the vocabulary words.

As part of the closing conversation, we included a goodbye gift task. The experimenter brought out a tray with several objects on it: a small toy frog (because the frog was present in the robot's story), a small book (because the robot expressed great interest in stories), a sticker of the robot's favorite color (blue), and an orange sticker. The child could pick an object to give to the robot, and the experimenter followed up by asking why the child had picked that gift.

After the robot interaction, the experimenter administered a receptive vocabulary test of the six target words in the story. For each word, four pictures taken from the story's illustrations were shown to the child. The child was asked to point to the picture matching the target word. We examined both children's receptive knowledge of the words as well as children's expressive or productive abilities during the story retelling, since children who can recognize a word may or may not be able to produce it themselves.

This was followed by the Inclusion of Other in Self task, adapted for children as described in Kory-Westlund et al. (2018). In this task, children are shown seven pairs of circles that proceed from not overlapping at all to overlapping almost entirely. They are asked to point to the circles showing how close they feel to five different entities: their best friend, their parent, a bad guy they saw in a movie, their pet (or if they have no pet, their favorite toy), and the robot. These five entities were included because we were curious how children might rate the robot compared to other people and things they might feel close to.

Then the experimenter asked several questions taken from the Social Acceptance Scale for Kindergarten Children (Favazza and Odom, 1996; Favazza et al., 2000) regarding how accepting children might be of the robot and its hearing difficulties, as well as of other children who might have hearing difficulties, as described in Kory-Westlund and Breazeal (2019). Finally, children performed a Picture Sorting Task (Kory-Westlund and Breazeal, 2019), in which they were asked to arrange a set of eight entities along a line. The entities included a baby, a frog, a cat, a teddy bear, a computer, a mechanical robot arm, a robot from a movie (e.g., Baymax, WALL-e, or R2D2, depending on which the child was familiar with), and Tega. The line was anchored at one end with a picture of an adult human female and at the other with a picture of a table. We wanted to see where children placed the robot in relation to the other entities.

### 3.6. Materials

We used the Tega robot, a colorful, fluffy squash and stretch robot designed for interactions with young children (Kory Westlund et al., 2016a) (see Figure 1). The robot is covered in red fur with blue stripes and uses an Android phone to display an animated face and run control software. The face has blue oval eyes and a white mouth, both of which can change shape to display different facial expressions and mouth movements (visemes) during speech. The robot can move up and down, tilt sideways, rotate from side to side, and lean forward and backward. The experimenters referred to the robot by name (not with pronouns) in a non-gendered way throughout the study.

Speech was recorded by a human adult female and shifted to a higher pitch to sound more child-like. All robot speech was sent through the automated audio entrainment module and streamed to the robot. For the *Entrainment* conditions, all speech was entrained; for the *No Entrainment* conditions, processing still occurred, but the speech simply passed through and was not changed. The reason for this was to incur the same delay (generally a latency of less than 1–2 s) that results from entraining and streaming speech in both conditions. More details regarding entrainment are provided below.

We used a Google Nexus 9 8.9-inch tablet to display the story. Touchscreen tablets have effectively engaged children and social robots in shared tasks (Park et al., 2014), including storytelling activities (Kory and Breazeal, 2014; Kory Westlund et al., 2017b). We used the same custom software on the tablet to display the story pages as in Kory Westlund et al. (2017b), which allowed the teleoperator to turn the pages at appropriate times. This software is open-source and available online under the MIT License at https://github.com/mitmedialab/SAR-opal-base/.

### 3.7. Teleoperation

As in the prior study (Kory Westlund et al., 2017b), we used custom teleoperation software to control the robot and digital storybook. The teleoperation software is open-source and available online under the MIT License at https://github.com/mitmedialab/tega_teleop/. The experimenters were all trained to control the robot by an expert teleoperator.

Using teleoperation allowed the robot to appear autonomous while removing technical barriers, primarily natural language understanding, since the teleoperator could be in the loop to parse language. The teleoperator triggered when the robot began each sequence of actions (speech, physical motions, and gaze), and when the storybook should turn the page. Thus, the teleoperator had to attend to timing in order to trigger action sequences at the right times. The timing of actions within sequences was automatic and thus consistent across children. There were also several occasions when the teleoperator had to listen to children's speech and choose the most appropriate of a small set of different action sequence options to trigger, namely during the picture conversation task.

The teleoperator performed one of two actions if the child asked an unexpected question or said something unusual. During the conversation portion of the interaction, the teleoperator could trigger one of the generic responses (e.g., “Mmhm!,” “Hm, I don't know!”) in reply. During the remainder of the interaction, the teleoperator had to continue in accordance with the interaction script, which essentially ignored unexpected behaviors. While this is not ideal from an interaction standpoint, it was necessary to ensure reasonably consistent behavior on the part of the robot across children.

### 3.8. Entrainment

In the *Entrainment* condition, the speaking rate and pitch of the robot's voice were automatically adjusted to be more similar to the child. In addition, the robot's volume and exuberance were manually adapted by the teleoperator.

For speaking rate and pitch entrainment, the child's speech was automatically collected via the robot's microphone when it was the child's turn to speak in the conversation. Using automatic software scripts with Praat (audio analysis software), various features of the children's speech were extracted and used to modify the robot's recorded speech files. These modified audio files were then streamed to the robot for playback.

For speaking rate, the robot's speech was sped up or slowed down to match the child's speaking rate. Thus, if a child spoke slowly, the robot slowed down its speech as well. We included ceiling and floor values such that the robot's speech would only ever be sped up or slowed down by a maximum amount, ensuring that the speech stayed within a reasonable set of speeds. We used the Praat script for speaking rate detection from de Jong and Wempe (2009). The code for our entrainment module is open-source and available online under a GNU General Public License v3.0 at https://github.com/mitmedialab/rr_audio_entrainer/.

The mean pitch of the robot's speech was shifted up or down. In doing this, the robot matches two features: (1) the child's age, (2) the child's current mean pitch. In general, people speak at a particular fundamental frequency, but there is variation within an individual (pitch sigma). Thus, we provided a table of mean fundamental frequencies for different age children based on the values computed in prior work (Weinberg and Zlatin, 1970; Bennett, 1983; Sorenson, 1989; Hacki and Heitmüller, 1999; Baker et al., 2008; Gelfer and Denor, 2014). For a given child, all of the robot's speech was first shifted to have the mean pitch for children of that age. Then, since an individual may vary their pitch in each utterance, the pitch of each utterance was also shifted up or down slightly based on whether the child's most recent utterance was higher or lower. Unlike Lubold and colleagues (Lubold et al., 2016, 2018), we did not adapt the pitch contour of the robot's speech. Because the base sounds for the robot's speech were recorded by a human (not flat text-to-speech as in Lubold et al.'s work), the sounds had their own pitch contours. Pilot tests showed that morphing or replacing this contour led to speech that sounded unnatural (e.g., placing emphasis on the wrong syllables).

We also manually adapted the robot's volume and exuberance. During the introduction and first picture in the picture task, the teleoperator observed the child's behavior and personality: were they shy, passive, reserved, or quiet (less exuberant/quiet children)? Or were they loud, extroverted, active, smiley, or expressive (more exuberant/loud children)? Based on this binary division, the teleoperator adjusted the robot's audio playback volume twice, at two specific points during the interaction, to either be slightly quieter (for less exuberant/quiet children) or slightly louder (for more exuberant/louder children). Furthermore, the teleoperator triggered different animations to be played on the robot at six different points during the interaction—more excited and bigger animations for more exuberant/louder children; quieter, slower, animations for less exuberant/quieter children.

### 3.9. Data

We recorded audio and video of each interaction session using a camera set up on a tripod behind the robot, facing the child. All audio was transcribed by human transcriptionists for later language analyses. Children's responses to the posttest assessments were recorded on paper and later transferred to a spreadsheet.

### 3.10. Data Analysis

For the analysis of children's story retellings, we excluded the three 3-year-olds because one did not retell the story, and the other two needed extra prompting by the experimenter and were very brief in their responses. Of the remaining 83 children, one child's transcript could not be obtained due to missing audio data. Fifteen children did not retell the story (the number from each condition who did not retell the story was not significantly different). Thus, in total, we obtained story retell transcripts for 67 children (15 *E-B*; 9 *E-NB*; 22 *NE-B*; 21 *NE-NB*).

We analyzed children's transcribed story retells in terms of story length (word count), overall word usage, usage of target vocabulary words, and similarity of each child's story to the robot's original story. We created an automatic tool to obtain similarity scores for each child's story as compared to the robot's story, using a phrase and word matching algorithm. The algorithm proceeded as follows: First, take both stories (the original story and the child's story) and remove stopwords (i.e., words with no significant information such as “the,” “uh,” and “an”). Second, stem words—i.e., convert words to their original form. For example, “jumping” would be converted to “jump.” Third, find all N-grams in each story, where an N-gram is a continuous sequence of N words from both texts. Fourth, remove duplicate N-grams from one text. Fifth, count how many N-grams are the same in both texts. The number of matches is the similarity score. This algorithm produces a score reflecting the number of exact matching phrases in both stories—i.e., words used in the same order by both the child and robot. It also produces a higher match score for texts that have both more matching phrases and longer matching phrases. We also implemented an algorithm for counting similar matches that are close to each other, but not exactly the same. This algorithm was the same as the above, where the fifth step (counting matching N-grams) used a fuzzy string matching algorithm to determine if the N-grams matched.

When running the algorithm to match stories, we used *N* = *3* for computing exact match scores because a smaller *N* may not retain enough information to be considered actual phrase matching, while a larger *N* may encompass more information than would constitute a single phrase. For determining similar match scores, we used *N* = *4*, so that when phrases differed by one word, or used a different word in the middle of a similar phrase, they might still match, as would be expected for similar phrases. We combined the exact and similar match scores to get a single overall similarity score for each child's story that reflected the child's overall use of exact and similar matching phrases.

For example, the robot's story included the sentences, “The baby frog liked the boy and wanted to be his new pet. The boy and the dog were happy to have a new pet frog to take home.” After stopword removal and stemming, this was converted to: “baby frog like boy want be new pet boy dog happy new pet frog take home.” One child's story included the similar section, “Then he hopped on his hand and he wanted to be his pet. And then the dog and the boy was happy to have a new pet,” which was converted to: “hop hand want be pet dog boy happy new pet.” There were several exactly matching phrases, e.g., “*happy new pet.”* There were also several similar matching phrases, e.g., (robot) “*be pet boy dog”*/(child) “*be pet dog boy.”*

We obtained children's facial expressions from the recorded videos using Affdex, emotion measurement software from Affectiva, Inc., Boston, MA, USA (McDuff et al., 2016). Affdex can detect 15 facial expressions, which are used to detect whether the face is displaying nine different affective states. Affdex only recognizes outward expressions of affect (i.e., facial configuration patterns), which does not imply detecting any underlying feelings or inferring deep internal states (though they are believed to be correlated). For each frame of a video, Affdex attempts to detect a face. If a face is detected, Affdex scores each affective state as well as the presence of each expression in the range 0 (no expression/affective state detected) to 100 (expression or state fully present); middle values represent an expression or state that is partially present. However, these values are relative and Affdex does not specify what the exact difference between scores means. For more detail on the algorithms used for facial affect classification, see Senechal et al. (2015). We analyzed affect data for 74 children (16 *E-B*; 11 *E-NB*; 26 *NE-B*; 21 *NE-NB*). For the remaining 12 children, little or no affect data were collected as a result of system failures, such as children's faces not being recognized by Affdex.

We focused our analysis on the following affective states and facial expressions: joy, fear, sadness, surprise, concentration, disappointment, relaxation, engagement, valence, attention, laughter, and smiles. We included valence in addition to specific emotions such as joy because Affdex uses different sets of facial expressions to detect the likelihood that a face is showing each affective state. Thus, valence is not detected from, e.g., the emotions joy or sadness; instead, it is calculated from a set of facial expressions that is somewhat different than, though overlapping with, the set of expressions used to calculate other emotions. The expression “concentration” was called “contempt” by Affectiva. Affectiva has no label for concentration or thinking expressions. Affectiva uses brow furrows and smirks to classify contempt; prior work has found that brow furrowing and various lip movements present in smirks such as mouth dimpling and lip tightens are also associated with concentration (Oster, 1978; Rozin and Cohen, 2003; Littlewort et al., 2011). Furthermore, contempt is generally defined as “the feeling that a person or thing is worthless or beneath consideration,” which, as in Kory Westlund et al. (2017b), did not make sense in this context; children's expressions were more indicative of concentration.

We coded children's responses to the Social Acceptance Scale questions on a 3-point scale, with “*no”* as 0, “*maybe”* as 1, and “*yes”* as 2. We labeled children's placement of the entities in the Picture Sorting Task, with the anchor on one end (the human) at position 1 and the anchor at the other (the table) at position 10. Thus, a lower rank indicated that children placed the entity closer to the adult woman. We counted positions to determine what rank was held by each picture. We also computed scores for Tega's rank relative to the other entities. For example, we subtracted the human baby's rank from Tega's rank to get Tega's rank relative to the human baby and human adult. Because Tega's position among the entities was dependent on where children placed the other entities in the task, we examined where children placed all the different entities.

We coded whether children agreed to do the fourth picture and whether they gave the robot their sticker with “*no”* as 0 and “*yes”* as 1. We coded children's selections in the goodbye gift task as follows: *frog* as 4, *book* as 3, *blue sticker* as 2, and *orange sticker* as 1. We also coded the comments children made regarding why they selected a particular gift with the following rubric: 2 if they referenced the robot or the robot's feelings (e.g., “Tega would like it because frog jumped out in story,” “Tega likes books,” “Because he wanted a sticker”); 1 for a somewhat relevant comment, mentioning the interaction (e.g., “It was in the story”); 0 for no explanation, reference to themselves, or an irrelevant comment (e.g., “It is swamp week at camp,” “I don't know”).

## 4. Results

Our results are divided below into two parts, each reflecting one of our hypothesis areas: (1) *Learning*: We asked whether the robot's entrainment and backstory would increase children's learning with the robot and emulation of the robot's story; and (2) *Rapport, relationship, and social behavior:* We asked whether children would show greater rapport, acceptance, positive emotion, engagement, and closeness to the robot as a result of its entrainment and backstory.

### 4.1. Learning (H1, H2, H3)

For all learning-related analyses of variance, we included Age as a covariate because we expected that children's age would be related to their language ability and thus to their vocabulary scores and the complexity and/or length of their stories.

#### 4.1.1. Target Vocabulary Word Identification (H1)

**Figure 2:**
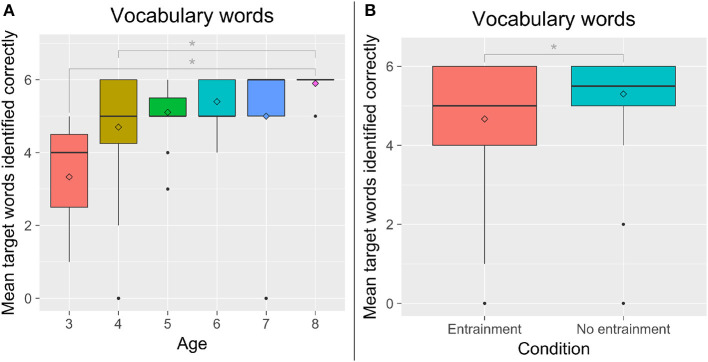
**(A)** The number of words correctly identified by children of each age group. **(B)** The number of words correctly identified by entrainment condition. ^*^p < 0.05.

We performed 2 × 2 between-subjects analyses of variance with Entrainment (*E* vs. *NE*) and Backstory (B vs. NB) with Age as a covariate. We found a significant effect of Age on the total vocabulary words identified correctly, *F*_(5, 77)_ = 2.76, *p* = 0.024, $\eta_{p}^{2}$ = 0.15. Eight-year-olds correctly identified the most words, while 3-year-olds correctly identified the least (Figure 2A). We also found a significant effect of Entrainment on children's identification of the target words, *F*_(1, 77)_ = 5.47, *p* = 0.022, $\eta_{p}^{2}$ = 0.07. Contrary to our hypotheses, children in the *NE* condition correctly identified more words than children in the *E* condition; however, in both conditions, there appeared to be a ceiling effect (Figure 2B). Older children were more likely to correctly identify words than younger children, r_*s*(85)_ = 0.367, *p* < 0.001.

#### 4.1.2. Target Vocabulary Word Use (H2, H3)

**Figure 3:**
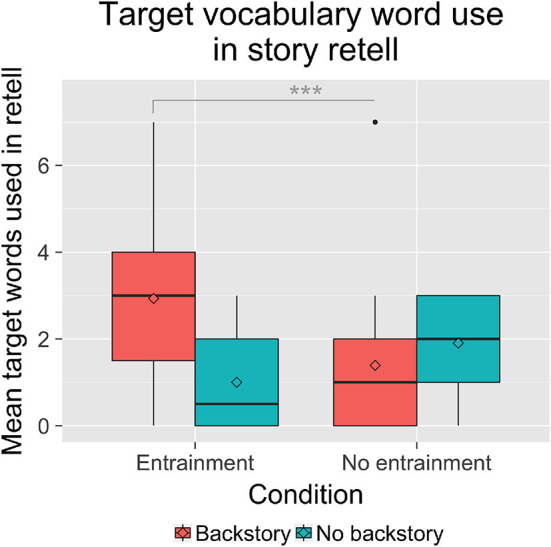
Children in the *E,B* condition used more target words in their story retells than children in the other conditions. ^***^p < 0.001.

A 2 × 2 between-subjects analyses of variance with Entrainment (*E* vs. *NE*) and Backstory (B vs. NB) with Age as a covariate revealed a significant interaction between Entrainment and Backstory regarding children's use of the target vocabulary words in the story, *F*_(1, 59)_ = 9.45, *p* = 0.003, $\eta_{p}^{2}$ = 0.14. Children in the *E,B* condition used significantly more of the target words than children in all three other conditions (Figure 3).

Overall, we saw no correlation between children's recognition of words on the vocabulary test and their subsequent use of those words in their retells, r_*s*(67)_ = 0.047. However, there were trends showing that this did vary by condition, though none of the correlations were significant. If the robot entrained, children were more likely to use the words themselves if they had identified the words correct on the test, *E-B* r_*s*(15)_ = 0.253; *E-NB* r_*s*(10)_ = 0.254; children who did not receive entrainment were less likely to do so, *NE-B* r_*s*(23)_ = −0.077; *NE-NB r*_*s*(21)_ = 0.024.

In summary, given that children's scores on the vocabulary identification test were not significantly different by condition, these results suggest that the robot's entrainment and backstory did not impact children's initial encoding of the words, but did affect children's expressive use of the words in their retelling.

#### 4.1.3. Story Length (H3)

The robot's story was 435 words long, including the dialogic questions. The mean length of children's retells was 304 words (*SD* = 110.9). After stopword removal, the robot's story was 185 words, of which 99 were unique, non-overlapping words. The mean length of children's stories after stopword removal was 113 (*SD* = 41.7), with a mean of 63.1 unique words (*SD* = 19.0).

**Figure 4:**
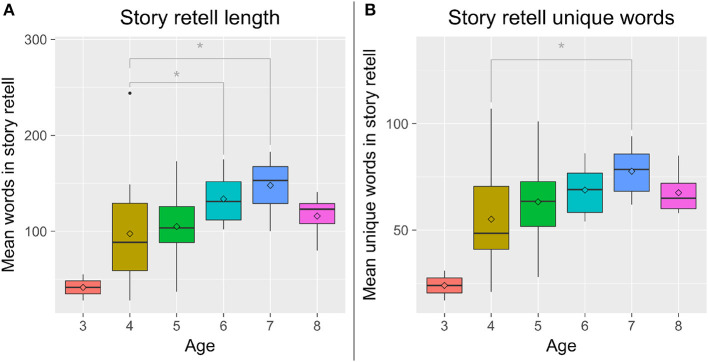
**(A)** Older children told longer stories than younger children. **(B)** Older children used more unique words than younger children. ^*^p < 0.05.

We performed 2 × 2 between-subjects analyses of variance with Entrainment (*E* vs. *NE*) and Backstory (*B* vs. *NB*) with Age as a covariate, which revealed a significant effect of Age on the length of children's stories after stopword removal, *F*_(4, 59)_ = 3.77, *p* = 0.008, $\eta_{p}^{2}$ = 0.20, and on the number of unique words children used, *F*_(4, 59)_ = 3.19, *p* = 0.019, $\eta_{p}^{2}$ = 0.17. *Post-hoc* tests revealed that 6- and 7-year-old children told longer stories than 4-year-old children, and 7-year-old children used more unique words than 4-year-old children (Figures 4A,B). The length of children's stories before stopword removal followed the same pattern, but was not statistically significant. This suggests that the primary difference between older (6–7 years) and younger (4–5 years) children's stories was their use of significant content words vs. stopwords.

#### 4.1.4. Mirroring the Robot's Story (H2, H3)

**Figure 5:**
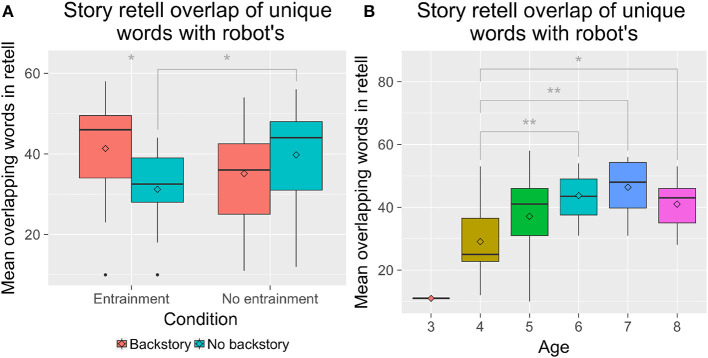
The number of overlapping words children used by entrainment condition **(A)** and by age **(B)**. ^*^p < 0.05; ^**^p < 0.01.

Children used a mean of 37.7 unique words (*SD* = 12.3) in their retells of the 99 unique words that the robot had used in its story. A 2 × 2 between-subjects analyses of variance with Entrainment (*E* vs. *NE*) and Backstory (*B* vs. *NB*) with Age as a covariate revealed that the number of overlapping unique words used was significantly different by Age, *F*_(4, 60)_ = 6.12, *p* < 0.001, $\eta_{p}^{2}$ = 0.29. We also observed a significant interaction of Entrainment with Backstory, *F*_(1, 60)_ = 6.42, *p* = 0.013, $\eta_{p}^{2}$ = 0.10. *Post-hoc* tests showed that older children overlapped more than younger children (Figure 5A). Children in the *E-NB* condition (*M* = 31.2, *SD* = 10.9) overlapped less than children in the *E-B* and *NE-NB* conditions (*E-B: M* = 41.3, *SD* = 13.2; *NE-B: M* = 36.2, *SD* = 10.6; *NE-NB: M* = 39.8, *SD* = 13.3) (Figure 5B).

Children's stories received mean scores of 41.3 (*SD* = 36.2) for their use of exact and similar phrases that mirrored the robot's phrases. However, we observed no significant differences between conditions in children's use of exact and similar matching phrases.

### 4.2. Rapport, Relationship, and Social Behavior (H4, H5, H6, H7)

#### 4.2.1. Acceptance of the Robot (H4)

**Figure 6:**
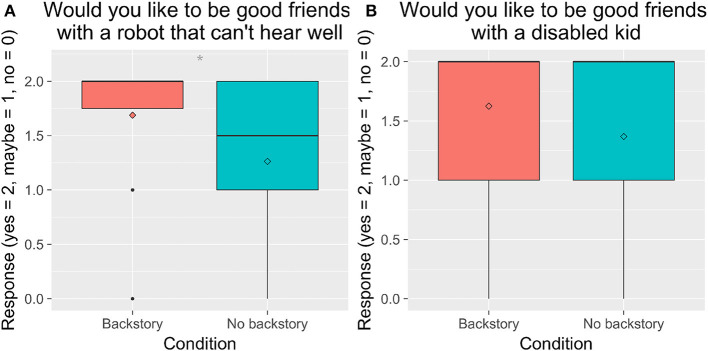
Children's responses to the question, “Would you like to be good friends with a robot who can't hear well?” and the question, “Would you like to be good friends with a handicapped or disabled kid?” by condition. ^*^p < 0.05.

We performed 2 × 2 between-subjects analyses of variance with Entrainment (*E* vs. *NE*) and Backstory (*B* vs. *NB*) for the questions asked about children's social acceptance of the robot and of other children. We found a significant main effect of Backstory of children's responses to the question “Would you like to be good friends with a robot who can't hear well,” *F*_(1, 82)_ = 7.55, *p* = 0.007, $\eta_{p}^{2}$ = 0.08. Children who heard the robot's backstory were more likely to respond positively than children who did not hear the robot's backstory. Children who heard the backstory were also somewhat more likely to respond positively to the question, “Would you like to be good friends with a handicapped or disabled kid,” though it was not statistically significant (Figure 6).

#### 4.2.2. Children's Expressivity and Positive Emotion (H5, H6)

Overall, children were highly attentive and engaged, and displayed surprise and other emotions during the story (see Table 2). To evaluate whether children showed greater engagement or positive emotion with the robot that entrained, we performed 2 × 2 between-subjects analyses of variance with Entrainment (*E* vs. *NE*) and Backstory (*B* vs. *NB*).

**Table 2:** Analysis of facial expressions during the interaction by condition.

**Expression**	**Overall**	**E-B**	**E-NB**	**NE-B**	**NE-NB**
Engagement	30.8 (11.7)	33.3 (13.3)	30.5 (12.0)	29.6 (11.2)	30.5 (11.4)
Attention	68.9 (13.4)	62.2 (21.1)	67.8 (15.2)	71.9 (5.56)	72.0 (9.51)
Valence	−0.738 (9.11)	3.51 (8.81)	5.75 (13.72)	−4.13 (5.20)	−2.72 (8.47)
Joy	7.13 (8.04)	9.13 (8.81)	12.1 (12.5)	5.48 (5.02)	5.61 (7.26)
Smiles	8.98 (8.82)	10.9 (9.35)	14.6 (13.4)	7.16 (5.65)	7.52 (8.31)
Laughter	0.13 (0.22)	0.23 (0.31)	0.28 (0.36)	0.08 (0.09)	0.07 (0.11)
Relaxation	3.53 (5.31)	4.13 (5.38)	6.63 (9.61)	2.49 (2.42)	3.06 (5.03)
Surprise	7.21 (6.96)	8.47 (9.22)	4.53 (4.63)	7.40 (5.32)	7.43 (7.84)
Disappointment	4.98 (3.98)	2.58 (2.01)	3.58 (3.03)	6.58 (4.37)	5.72 (4.05)
Fear	1.48 (2.06)	1.00 (1.40)	0.38 (0.66)	1.87 (2.04)	1.93 (2.72)
Concentration	2.92 (2.48)	2.02 (1.79)	2.11 (1.87)	3.20 (2.45)	3.72 (3.03)
Sadness	0.27 (0.46)	0.22 (0.34)	0.49 (0.54)	0.32 (0.59)	0.17 (0.24)

*Values can range from 0 (no expression present) to 100 (expression fully present), except Valence, which can range from −100 to 100. Each column lists mean and standard deviation*.

**Figure 7:**
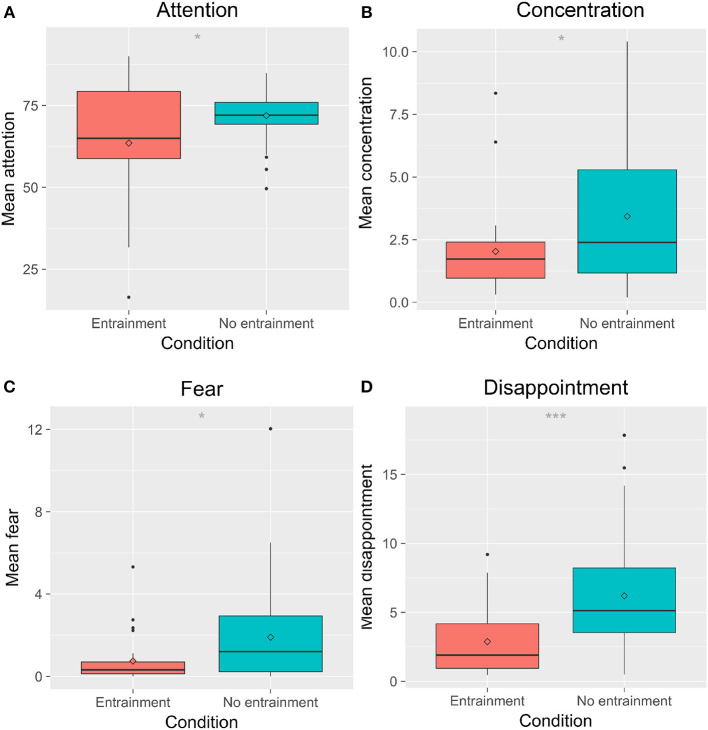
Children's overall negative affect varied by entrainment condition. **(A)** shows attention; **(B)** shows concentration; **(C)** shows fear; **(D)** shows disappointment. ^*^p < 0.05; ^***^p < 0.001.

**Figure 8:**
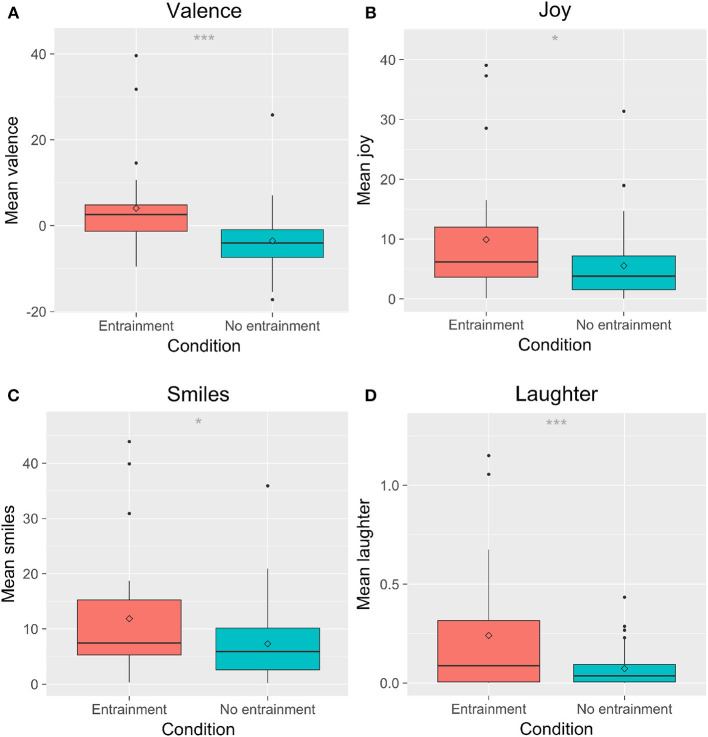
Children's overall postive affect varied by entrainment condition. **(A)** shows valence; **(B)** shows joy; **(C)** shows smiles; **(D)** shows laughter. ^*^p < 0.05; ^***^p < 0.001.

We found a significant main effect of Entrainment on children's expressions of joy, *F*_(1, 69)_ = 6.25, *p* = 0.015, $\eta_{p}^{2}$ = 0.070; fear, *F*_(1, 69)_ = 5.31, *p* = 0.024, $\eta_{p}^{2}$ = 0.074; concentration, *F*_(1, 69)_ = 5.09, *p* = 0.027, $\eta_{p}^{2}$ = 0.074; disappointment, *F*_(1, 69)_ = 12.7, *p* < 0.001, $\eta_{p}^{2}$ = 0.17; attention, *F*_(1, 69)_ = 5.66, *p* = 0.02, $\eta_{p}^{2}$ = 0.091; laughter, *F*_(1, 69)_ = 12.02, *p* < 0.001, $\eta_{p}^{2}$ = 0.13; smiles, *F*_(1, 69)_ = 5.82, *p* = 0.019, $\eta_{p}^{2}$ = 0.064; and valence, *F*_(1, 69)_ = 14.7, *p* = < 0.001, $\eta_{p}^{2}$ = 0.16. *Post-hoc* tests showed that children expressed less fear, concentration, disappointment, and attention in the *E* condition than in the *NE* condition (Figure 7). Children showed higher mean joy, laughter, valence (i.e., showed more affect with a positive valence), and more smiles in the *E* condition than in the *NE* condition (Figure 8). There were no significant differences in sadness, surprise, relaxation, or engagement; however, there was a trend for children in the *E* condition to show more relaxation than in the *NE* condition, which could have contributed to the higher valence seen in the *E* condition.

Next, we asked whether children's affect changed during the session. We split the affect data into the first half of the session and the second half of the session, using the data timestamps to determine the halfway point. We ran a 2 × 2 × 2 mixed ANOVA with time (within: first half vs. second half) × Entrainment (between: *E* vs. *N*E) × Backstory (between: *B* vs. *NB*). Although we hypothesized several changes in children's affect over time as a result of condition, we corrected for multiple comparisons here and only considered results significant when *p* < 0.004.

**Figure 9:**
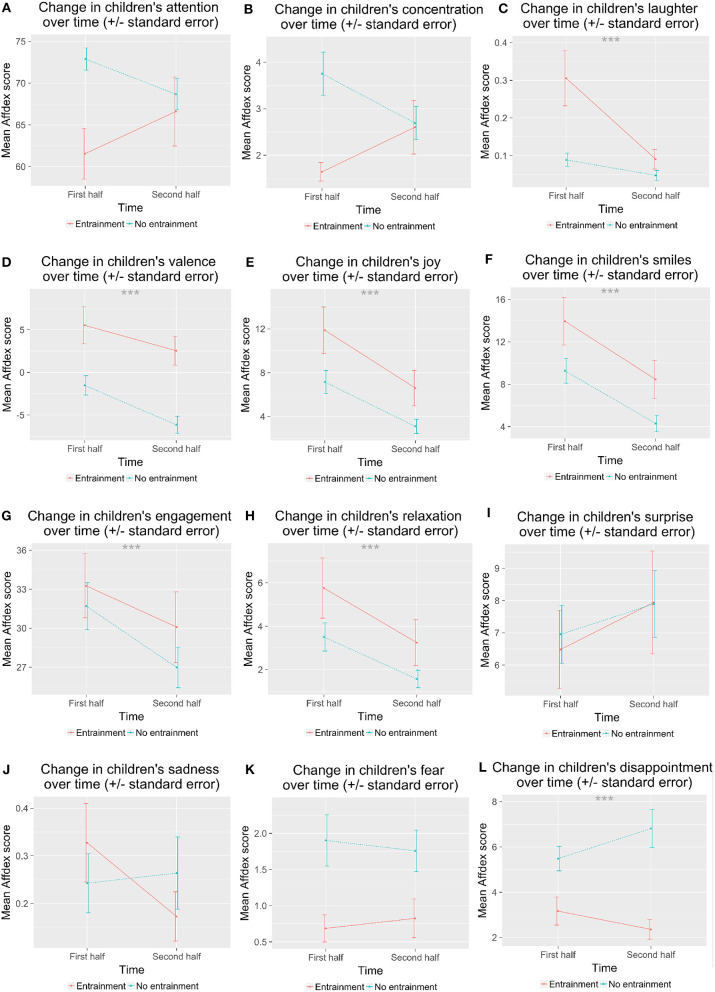
Children's affect during the first half and the second half of the interaction varied by entrainment condition. **(A)** shows attention; **(B)** shows concentration; **(C)** shows laughter; **(D)** shows valence; **(E)** shows joy; **(F)** shows smiles; **(G)** shows engagement; **(H)** shows relaxation; **(I)** shows surprise; **(J)** shows sadness; **(K)** shows fear; **(L)** shows disappointment. ^***^p < 0.001.

Like before, we found a significant main effect of Entrainment on disappointment, *F*_(1, 70)_ = 14.7, *p* < 0.001; laughter, *F*_(1, 70)_ = 8.94, *p* = 0.004; and valence, *F*_(1, 70)_ = 14.6, *p* < 0.001. There were trends for a main effect of Entrainment on joy, *F*_(1, 70)_ = 4.25, *p* = 0.043; fear, *F*_(1, 70)_ = 5.88, *p* = 0.018; attention, *F*_(1, 70)_ = 4.37, *p* = 0.040; and smiles, *F*_(1, 70)_ = 3.99, *p* = 0.0497. Children showed fewer expressions of fear and disappointment in the *E* than in the *NE* condition (Figure 9). Children showed more joy, more smiles, and higher valence in the *E* than the *NE* condition.

We found a significant main effect of time on joy, *F*_(1, 67)_ = 34.6, *p* < 0.001; valence, *F*_(1, 67)_ = 17.7, *p* < 0.001; engagement, *F*_(1, 67)_ = 10.3, *p* = 0.002; smiles, *F*_(1, 67)_ = 40.5, *p* < 0.001; relaxation, *F*_(1, 67)_ = 27.2, *p* < 0.001; laughter, *F*_(1, 67)_ = 11.9, *p* = 0.001. All of these decreased from the first half to the second half of the session.

We saw trends for interactions of Entrainment with time: concentration, *F*_(1, 67)_ = 6.79, *p* = 0.011; attention, *F*_(1, 67)_ = 5.47, *p* = 0.022; and laughter, *F*_(1, 67)_ = 7.82, *p* = 0.007. Children showed more concentration during the first half in the *NE* than in the *E* condition. Children showed more attention during the first half for *NE* vs. *E*, but they did not differ during the second half. Children laughed more in the first half in the *E* condition than in the *NE* condition, and decreased to the second half, while in the *NE* condition the amount of laughter did not change over time.

**Figure 10:**
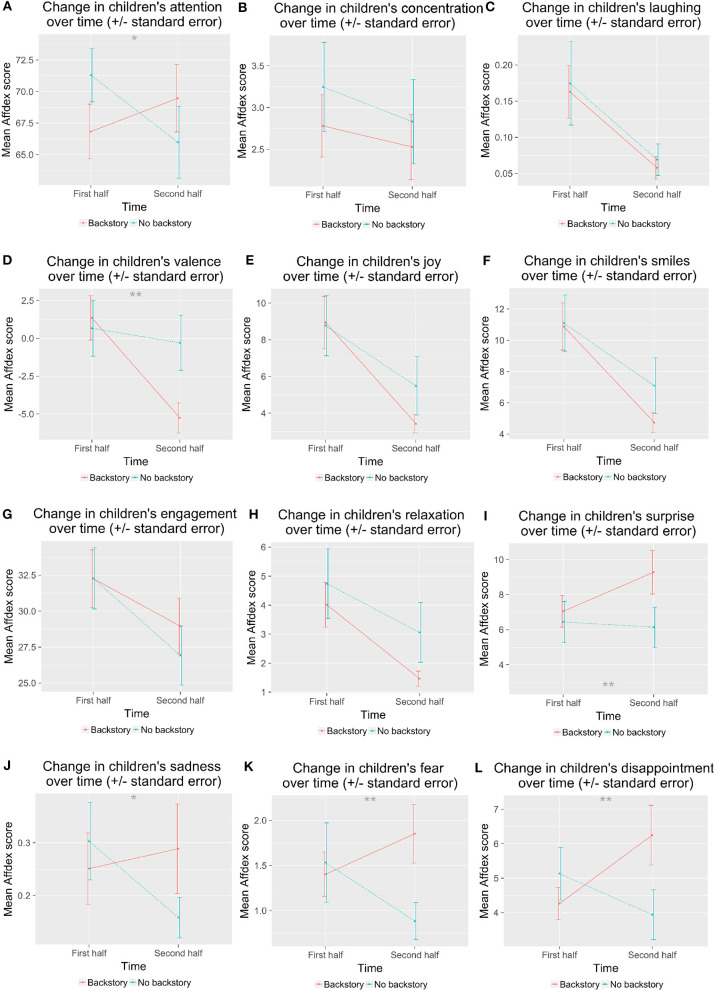
Children's affect during the first half and the second half of the interaction varied by backstory. **(A)** shows attention; **(B)** shows concentration; **(C)** shows laughter; **(D)** shows valence; **(E)** shows joy; **(F)** shows smiles; **(G)** shows engagement; **(H)** shows relaxation; **(I)** shows surprise; **(J)** shows sadness; **(K)** shows fear; **(L)** shows disappointment. ^*^p < 0.05; ^**^p < 0.01.

We also saw trends for interactions of time with Backstory for fear, *F*_(1, 67)_ = 8.55, *p* = 0.005; sadness, *F*_(1, 67)_ = 7.01, *p* = 0.010; disappointment, *F*_(1, 67)_ = 7.70, *p* = 0.007; attention, *F*_(1, 67)_ = 4.88, *p* = 0.031; and valence, *F*_(1, 67)_ = 8.12, *p* = 0.006 (Figure 10). Children expressed less fear in the second half of the session when they did not hear the backstory, but expressed somewhat more fear in the second half if they had heard the backstory. They expressed less sadness in the second half in *NB* condition, but did not change in *B* condition. Children's expressions of disappointment increased slightly in the *B* condition from first to second half, but not for the *NB* condition. Children's attention was higher initially in the *NB* condition and decreased slightly, while children's attention started lower in the *B* condition and increased slightly. Children showed decreased valence in the *B* condition from first half to second half, but not in the *NB* condition.

#### 4.2.3. Closeness to the Robot (H5, H6)

**Figure 11:**
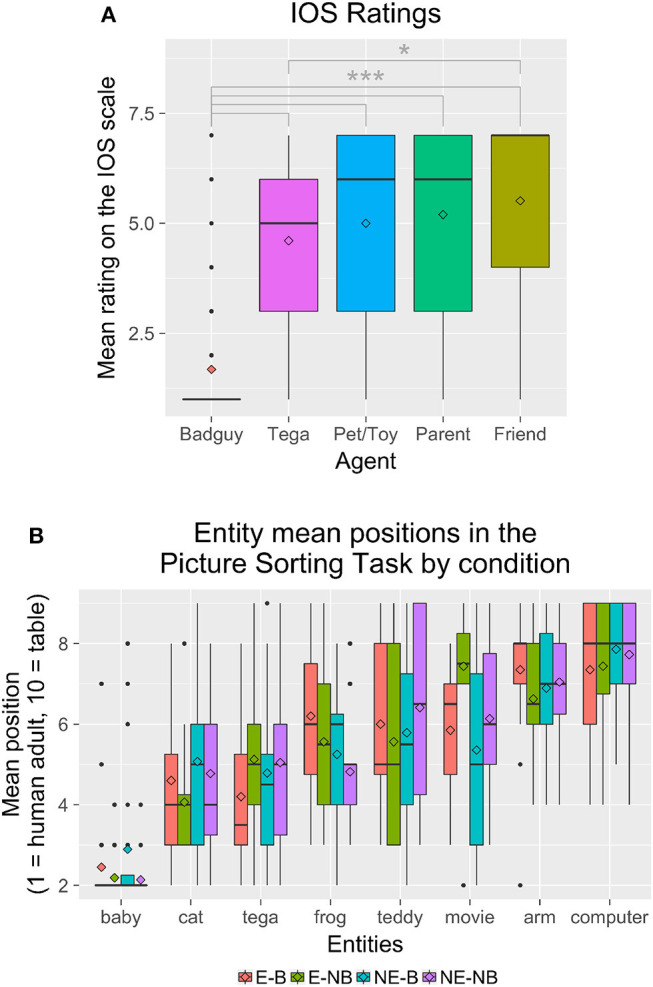
**(A)** Children's IOS ratings for each agent. **(B)** The mean position where children placed each entity in the Picture Sorting Task by condition. ^*^p < 0.05; ^***^p < 0.001.

We performed a 2 × 2 × 5 mixed ANOVA with Entrainment (*E* vs. *NE*) × Backstory (*B* vs. *NB*) × IOS agent (within: Friend, Parent, Tega, Pet/Toy, Bad guy). We found a significant effect of agent, *F*_(4, 302)_ = 61.9, *p* < 0.001. *Post-hoc* Tukey's HSD tests showed that the bad guy was rated significantly lower than all other agents. In addition, the robot was rated significantly lower than the friend, but was not significantly different from the parent or pet/toy (Figure 11A). Older children were more likely to rate Tega as closer, *r*_*s*(86)_ = 0.410, *p* < 0.001 (Figure 13A).

**Figure 12:**
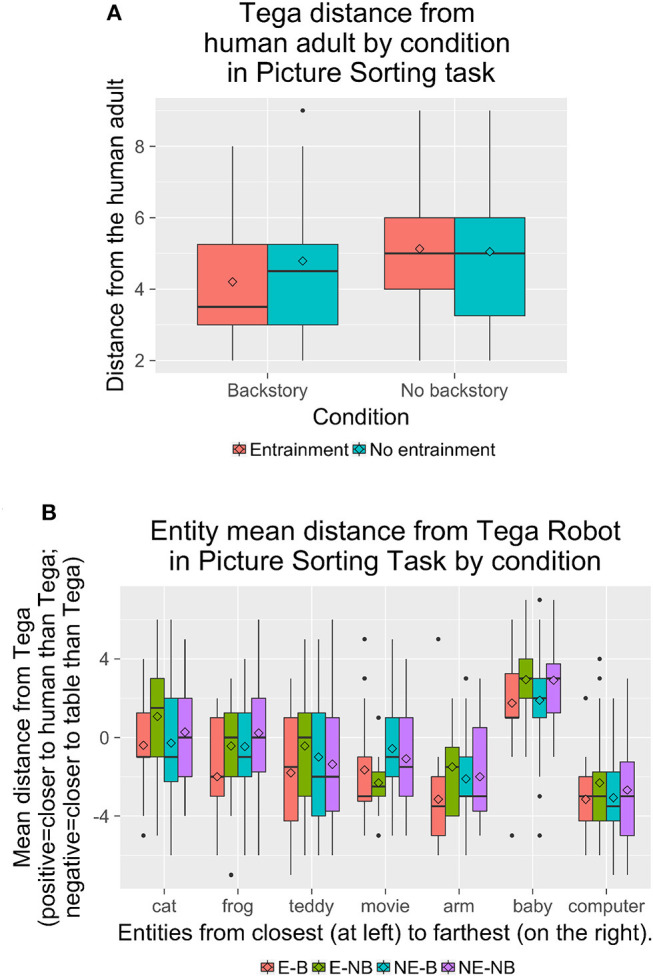
**(A)** Tega's mean distance from the human adult in the Picture Sorting Task by condition. **(B)** The distance of each entity from the Tega robot in the Picture Sorting Task by condition. There were trends for the Tega robot to be placed closer to the baby in the *B* condition than in the *NB* condition, closer to the movie robot in the *E* condition than in the *NE* condition, and closer to the frog in the *E-B* condition than in the other conditions.

Regarding the Picture Sorting Task, overall, Tega was placed at a mean position of 4.78 (*SD* = 1.80) (Figure 11B). Figure 12A shows results by condition for Tega's distance to the human, and Figure 12B shows the relative distance of each entity from the Tega robot by condition.

We performed a mixed ANOVA with Entrainment (between: *E* vs. *NE*) × Backstory (between: *B* vs. *NB*) × Entity (within: Tega robot, baby, cat, frog, teddy bear, movie robot, robot arm, computer) for the entity positions, as well as for the entity positions relative to the Tega robot. For entity positions, we observed a significant main effect of Entity, *F*_(7, 574)_ = 71.7, *p* < 0.001. We also observed a significant interaction of Entity with Entrainment, *F*_(7, 574)_ = 2.15, *p* = 0.037; and a significant interaction of Entity with Backstory, *F*_(7, 574)_ = 2.35, *p* = 0.022.

*Post-hoc* tests revealed that the baby was placed significantly closer to the human adult than all other entities. The cat was placed significantly closer to the human adult than all entities except for the Tega robot in the *E* condition, and closer to the human than all entities except Tega and the frog in the *NB* condition. In both the *NE* and *B* conditions, the cat was not placed significantly differently from Tega, the frog, movie robot, or teddy bear.

In the *E* condition, the Tega robot was significantly closer to the human adult than the robot arm, computer, movie robot, and teddy bear. It was farther from the human adult than the baby and was not placed in a significantly different position from the cat or frog. In the *NE* condition, Tega was only placed significantly closer to the human adult than the robot arm and computer; it was not placed significantly differently from the cat, frog, movie robot, or teddy bear. Tega was not placed in a significantly different position from the movie robot in the *B* condition, but was placed significantly farther from it (closer to the human) in the *NB* condition.

The frog was placed significantly closer to the human adult than the robot arm and computer, and significantly farther from the human adult than the baby, but otherwise its position did not differ significantly from any other entities, except in the *NB* condition, where it was placed closer than the movie robot.

In the *NE* condition, the robot arm was placed closer to the table than the frog and movie robot, but in the *E* condition, the robot arm was not placed significantly differently from the frog or movie robot. By Backstory, children in the *B* condition placed the robot arm closer to the table than all other entities except the computer and teddy bear, while in the *NB* condition the robot arm's position was also not signficantly different from the movie robot's. Finally, in the *NE* and *B* conditions, the computer was placed closer to the table than all entities except the robot arm, while in the *E* and *NB* conditions, the computer was also not significantly different from the movie robot.

Regarding the distance of each entity relative to the Tega robot, we observed a significant main effect of Entity, *F*_(6, 492)_ = 71.8, *p* < 0.001. We also observed a significant interaction of Entity with Entrainment, *F*_(6, 492)_ = 2.13, *p* = 0.049; and a trend toward an interaction of Entity with Backstory, *F*_(6, 492)_ = 2.11, *p* = 0.051. *Post-hoc* tests revealed that the baby was placed farther from Tega, and closer to the human adult than Tega was, than all other entities. There was a trend for children to place the Tega robot closer to the baby (and the baby closer to the human adult than Tega) in the *B* condition (mean difference = 1.83, *SD* = 2.55) than in the *NB* condition (*M* = 2.92, *SD* = 2.01).

The cat was placed closer to Tega than most other entities. It was not placed significantly differently than the teddy bear in the *E* condition; from the frog, movie robot, or teddy bear in the *NE* and *B* conditions; and from the frog in the *NB* condition.

The computer was placed farther from Tega than all entities except the robot arm and, in the *E* and *NB* conditions, the movie robot. The robot arm, in turn, was placed farther from Tega than all entities except the computer and teddy bear. In the *NB* and *NE* conditions, the robot arm was also not different than the movie robot; and in the *E* condition, the robot arm was also not different from the movie robot or frog. There was a trend for children to place Tega farther from the movie robot, and closer to the human than the movie robot was, in the *E* condition (*M* = −1.94, *SD* = 2.40) than in the *NE* condition (*M* = −0.80, *SD* = 2.69).

Finally, we also observed trends for Tega to be placed farther from the frog, and also closer to the human adult than the frog was, in the *E* (*E*: *M* = −1.31, *SD* = 2.77, *NE*: *M* = −0.16, *SD* = 2.62) and *B* conditions (*B*: *M* = −1.11, *SD* = 2.76, *NB*: *M* = −0.05, *SD* = 2.60).

We observed no significant differences between conditions regarding whether children were more likely to agree to do the fourth picture with the robot, give the robot their sticker in the sticker task, or give the robot a bigger goodbye gift (in terms of how meaningful the robot might think it to be). About half the children in each condition chose to do the fourth picture; we did not see any effects of the number of picture conversations (i.e., the three required vs. the optional fourth one) on the results. If we looked at children's likelihood to perform all three activities (adding up the fourth picture, the sticker, and the goodbye gift, rather than any one individually), we saw a trend for children in the *E-B* condition to be slightly more likely to do all three activities, though this was not statistically significant.

#### 4.2.4. Children's Mirroring, Learning, and Relationship (H7)

**Figure 13:**
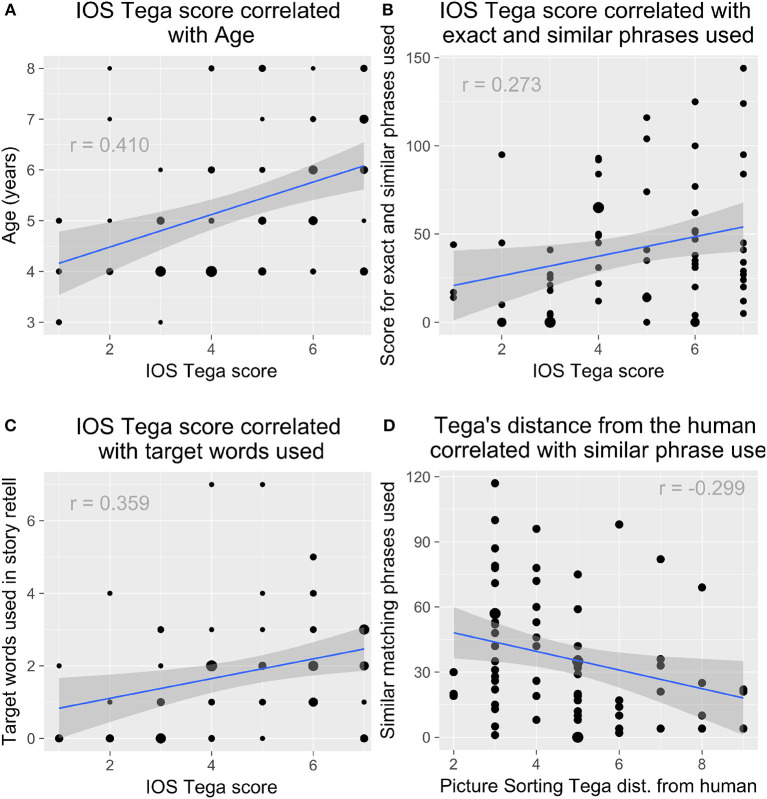
**(A)** Older children rated the robot as closer in the IOS task. Children who rated the robot as closer were more likely to **(B)** use the target words in their stories and **(C)** emulate the robot's phrases. **(D)** Children who placed the robot closer to the human in the Picture Sorting Task were also more likely to emulate the robot.

We found that children who gave Tega a closer score on the IOS task were also more likely to use the target words in their stories, *r*_*s*(67)_ = 0.359, *p* = 0.003 (Figure 13C). They were also more likely to emulate the robot's stories as reflected by the number of exact and similar phrases used in their retells, *r*_*s*(67)_ = 0.273, *p* = 0.025 (Figure 13B). Given that age also correlated with children's ratings of Tega on the IOS task, we might suspect that age is more relevant than how close children felt to the robot. However, age did not correlate with children's use of exact and similar phrases, which suggests a deeper story.

In addition, children who placed Tega closer to the human in the Picture Sorting Task were also more likely to use phrases similar to the robot's, *r*_*s*(67)_ = −0.299, *p* = 0.014 (Figure 13D). There was a trend for children who placed Tega closer to the human to also rate Tega more closely on the IOS task, *r*_*s*(86)_ = −0.197, *p* = 0.069.

We did not observe any significant correlations of children's vocabulary scores with their phrase mirroring or any of the relationship assessments.

## 5. Discussion

We asked whether a social robot that entrained its speech and behavior to individual children and provided an appropriate backstory about its abilities could increase children's rapport, positive relationship, acceptance, engagement, and learning with the robot. Below, we discuss the main findings and then discuss the implications of these findings.

### 5.1. Learning

Children learned the target vocabulary words in the robot's story and were generally attentive and engaged with the robot regardless of the experimental condition. They showed a variety of emotional expressions throughout the interaction. Children remembered the robot's story as evidenced by their ability to retell the story and their identification of target words on the vocabulary test. These results are in line with the prior study using this story activity (Kory Westlund et al., 2017b), which found significant learning gains.

We did see differences in children's learning by condition. Contrary to our hypotheses (H1), children in the *No Entrainment* condition correctly identified more target words than children in the *Entrainment* condition (Figure 2B). This could be for several reasons. A prior study found that a robot tutor that employed social adaptive behaviors led to lower learning gains than a robot that did not act as socially (Kennedy et al., 2015). Thus, perhaps the entraining robot was perceived more socially, which was detrimental in learning. This is contrary to our hypotheses regarding the importance of social behavior, rapport, and relationship in language learning with peers. However, in the prior study, children performed a math task with the robot tutor. The authors hypothesized that perhaps children were paying attention to the robot's social behavior as opposed to the lessons it was providing, or, alternatively, that the social behavior placed greater cognitive load on children thus inhibiting their ability to perform in the math task. Performance on a math task in a tutoring format may indeed benefit less from a robot's social behaviors than performance in a language-based story activity in a peer-learning format.

A second explanation pertains to the learning results we observed. There was a ceiling effect and little variance in children's responses, with 43% of children correctly identifying all six target words, and 41% correctly identifying 5 of the target words. If a significant number of children were already familiar with the target words, then the vocabulary tests would not reflect their learning during the task with the robot; the difference between conditions may not reflect children's learning in the task. Furthermore, given that children's receptive language abilities may precede their expressive abilities (Bloom, 1974; Ingram, 1974; Sénéchal, 1997), we would expect that children who correctly identified more words to also use more of them in their stories (H2), reflecting greater understanding and deeper encoding of the words (this was also seen in the prior study, Kory Westlund et al., 2017b). However, we did not see this correlation: children's use of the target words was not significantly correlated with correct identification of the words. In fact, children's use of the target words was significantly greater in the *E-B* condition than all others, in line with our hypotheses (H3) (Figure 3). Additionally, while the patterns were not significant, children were moderately more likely to use the words if they had identified them correctly in the *Entrainment* condition than in the *No Entrainment* condition. These results suggest that the robot's rapport- and relationship-building behaviors affected either or both of (a) children's learning and deeper understanding of the words such that they were more able to expressively use the words, or (b) children's mirroring of the robot's speech such that they used more of these target words, both of which would be in line with prior work linking rapport to learning (Sinha and Cassell, 2015a,b). This was also a short-term encounter. Given the positive aspects we see here regarding word use and mirroring, we expect that over multiple sessions, we would see greater differences in word learning.

When we examined children's mirroring of the robot's speech, we saw that children did mirror the robot (H2, Figures 3, 5), in line with past work suggesting that children may mirror adults' syntax and speech (Huttenlocher et al., 2004) and earlier work in human-computer interaction showing that adults will entrain to computers and robots (e.g., Pearson et al., 2006; Lubold et al., 2018). However, we saw no significant differences in children's emulation of the robot's phrases, and in fact, less overlap in the number of unique words used by children that mirrored the words the robot used in the *E-NB* condition, and little difference among the other conditions (contrary to H3). This suggests that perhaps entrainment did not affect children's mirroring of the words the robot used so much as their expressive ability to use the key words present in the story. Prior work has shown that social robots can be successful at prompting children to demonstrate expressive vocabulary skills in both vocabulary test and storytelling contexts (e.g., Kory and Breazeal, 2014; Kory Westlund et al., 2017b; Wallbridge et al., 2018). The present study suggests that the robot's entrainment may influence expressive ability.

The lack of difference in phrase mirroring was counter to our hypotheses (H3). Perhaps children did not feel sufficiently more rapport with the entraining robot for this to affect their storytelling. Indeed, in all conditions, the robot was a friendly, expressive character, which children generally said they felt close to—as close as to pet or parent, though less close than to a best friend. The entrainment only affected the robot's speech and some animations (which were played primarily in accompaniment with speech). In particular, if a child was very shy and rarely spoke, then the robot had fewer opportunities to adapt and entrain to that child. Perhaps greater difference would be seen if the robot also entrained other behaviors, such as posture, gesture, or word use. Another explanation is that perhaps language mirroring is not as closely linked to rapport as we expected; there is limited research so far suggesting this link, and more is needed.

### 5.2. Rapport, Relationship, and Social Behavior

The robot's entrainment and backstory also affected children's displays of positive emotions during the interaction. All children were engaged, but children in the *E-B* condition showed more positive emotions (e.g., joy, laughter, smiles, and positive valence), as well as fewer negative emotions (e.g., disappointment, fear) (supporting H5 and H6; see Figures 7–10). Laughter and smiling are social behaviors (Provine, 2001; Smidl, 2006; Manson et al., 2013). We also saw trends for children to be more helpful and accommodating in the *E-B* condition, as one might expect with a more social agent (Reeves and Nass, 1996), as evidenced by their behavior with fourth picture, the sticker task, and the goodbye gift. This is evidence that the robot's entrainment and backstory improved children's enjoyment of the interaction and may have perceived it as more of a social agent, perhaps a result of increased rapport (supporting H5 and H6).

Children in the *E-B* condition also showed fewer attentive expressions, though only during the first half of the interaction (they did not differ later on). This could mean that these children were in fact less attentive initially, or it could mean that they were showing more positive attentive expressions that were coded by the affect recognition software as engagement and joy. If they were less attentive, we might expect this to be reflected in their vocabulary scores and story retellings—perhaps this is why these children did not identify as many words correctly. However, children in the *E-B* condition showed just as many expressions of engagement as children in the other conditions, were just as likely to retell the story, and as noted earlier, there were few significant differences by condition in children's story retellings beyond *more* use of the target words by children in the *E-B* condition. An alternative explanation is that perhaps children's attentive looks were related to how much cognitive effort was involved in performing the task. The robot's entrainment and backstory could have improved rapport and made the interaction more fluent, easier, and smoother, thus requiring less intense attention by children. This would be especially apparent earlier in the interaction, immediately following the robot's backstory disclosure and during the picture conversation task, when the robot was entraining more frequently due to the increased number of conversational turns during that task.

Related to this, we saw that children's attention increased over time in the *B* condition, but decreased in the *NB* condition, while multiple negative emotions (fear, disappointment, sadness) were displayed more frequently over time in the *B* condition than in the *NB* condition. For all other affective states measured, the change over time was not significant, though there were patterns for decreases in positive affect (e.g., joy, smiles, etc.) over time for all children. If children's attentive expressions were related to cognitive effort, this could indicate that in the *B* condition, children felt that over time, they had to attend more carefully to the robot (putting in more effort) in order to help it and deal with its hearing limitations. This could, perhaps, have led to increased feelings of difficulty interacting with the robot over time, which could have led to the increased displays of negative emotions that we observed in the *B* condition.

Regarding the decrease in attention in the *NB* condition, it may be that these children became less attentive because they were growing bored or were not as invested in the interaction. Indeed, while not statistically significant, children's engagement did decrease slightly more over time in the *NB* condition than in the *B* condition. There were also no affective states for which children in the *NB* condition increased their expression over time, suggesting that they became less expressive overall, which may be indicative of boredom or less emotional investment in the interaction.

We observed that children showed greater acceptance of the robot when they had heard the robot's backstory, as we expected (H4; Figure 6). Children's increased negative affect seen in the *B* condition may also reflect increased sympathy for the robot. Regardless, it seems that the robot's story influenced children's perceptions of it, in line with prior work showing that a robot's story does influence how people understand and react to it (Stenzel et al., 2012; Klapper et al., 2014; Darling et al., 2015; Kory Westlund et al., 2016b). Interestingly, this effect seemed to carry over to children's ideas about being friends with other children. While only a trend, it suggests room for future interventions using robots to help children understand and accept others different from themselves.

As noted above, children generally felt as close to the robot as they did to a pet, favorite toy, or parent, though not quite so close as to their best friend (Figure 11A). They generally placed Tega closer to the human adult than the table in the Picture Sorting Task, and frequently close to the human baby and to the cat (Figures 11B, 12). These results present an intriguing picture regarding children's perceptions of the robot as a peer- or friend-like, non-human, animate entity. Children did not confuse the robot with a human; they knew it was different. Children seemed to clearly find companionship in the robot and to place it in a category between friend, pet, and authority figure. It was not merely a machine or computer; it was seen as more animate and alive—but not in the same category as a human. This jibes with prior work suggesting that children may categorize robots as in-between entities, with attributes of both living beings and mechanical artifacts (Kahn et al., 2002, 2012; Severson and Carlson, 2010). Perhaps children observed that some of the things that are messy about human relationships, such as the kinds of conflict that arise and the emotions that others display, are not the same in robot relationships—perhaps they are more like pet relationships. In this case, the robot did not get overly upset when it did not receive the sticker it wanted in the sticker task; it was generally cheerful throughout the interaction, which perhaps would not have been the case with another child. It is also likely that the robot's morphology influenced children's perceptions, since the robot we used was fluffy, colorful, and moved more like an animated character or sidekick than a humanoid being.

In support of our hypotheses regarding the connection between children's feelings of closeness, rapport, and relationship with learning and mirroring the robot (H7), we observed that children who rated the robot as closer to themselves also used the target words more often and emulated the robot's story more (Figure 13). This is in line with earlier work linking rapport to learning (Sinha and Cassell, 2015a,b). However, we also saw that age correlated with children's ratings of Tega on the IOS task. Older children rated the robot as closer; younger children as less closer. Perhaps younger children were less sure of the robot and needed more time to become comfortable with it. Given these correlations, we might suspect that age was more relevant to children's use of the target words and emulation of the robot's story than children's closeness ratings. However, children's age did not correlate with children's emulation of the robot's phrases at all, which suggests that this emulation was in fact related to children's feelings of closeness.

Finally, we also observed a few age differences. The length of children's story retellings differed with respect to their age, but did not vary by condition (Figure 4). Notably, the stories told by 6- and 7-year-old children were longest. The stories of 8-year-old children were not quite so long, which may have been because they were less interested in the story, rather than less capable. The story and activity were designed with 4–7-year-olds in mind. The story may have been a little on the difficult side for the younger children, and on the easy side (and thus perhaps a little boring) for the oldest. However, even the children outside the target age range for the activity were receptive to the social robot, showing engagement, learning, and emulation.

Taken together, these results show that the robot's rapport and relationship-building behaviors do matter in interactions with young children. A robot that deliberately emulates a child's speech in a way similar to how people mirror each other can elicit more positive emotion and greater emulation of key words in a language learning activity. Children's feelings of closeness are related to their emulation of the robot's words in their stories.

### 5.3. Relation to Related Work

Our results also mirror, to an extent, the results in the prior study that explored a robot's use of expressive vs. flat speech (Kory Westlund et al., 2017b). In both studies, the robot's entrainment, backstory, and expressivity reflected the sensitivity the robot showed to the interaction. This sensitivity influenced children's engagement and learning. This is in line with work examining nonverbal behaviors in human-human learning interactions, in particular, nonverbal immediacy. *Nonverbal immediacy* refers to the *perceptual availability* of one's interaction partner, i.e., the use of nonverbal behaviors including gaze, gesture, posture, facial expressions, and vocal qualities such as prosody to signal general responsiveness and attentiveness. In human-human learning interactions, nonverbal immediacy has been linked to increased learning gains (Mehrabian, 1968; Christophel, 1990; Witt et al., 2004). When we examine prior child-robot interaction studies, we see that they have found a similar pattern of results to these human-human studies: The use of nonverbal immediacy behaviors including socially contingent behavior, appropriate gaze and posture, and vocal expressivity increased children's learning, engagement, and trust in a learning companion (Breazeal et al., 2016a; Kennedy et al., 2017; Kory Westlund et al., 2017a,b). Thus, it may be that the entrainment behaviors used by the robot increased its perceived immediacy and perceived sensitivity to the interaction.

However, in other work on language learning with social robots, the robot's social interactive capabilities have been found to influence children's relationships and social acceptance of the robot, but not their learning (e.g., Kanda et al., 2004, 2007, 2012). Indeed, some work has shown no significant differences in children's word learning from a social robot (with numerous embodied social capabilities) than from a tablet (e.g., Kory Westlund et al., 2015; Vogt et al., 2019). Arguably, these studies suggest a contrary story in which the robot's social capabilities may not affect children's learning that much.

These studies, however, have generally included learning tasks that did not require a robot or much social behavior for learning to proceed. For example, the second language learning activities used by Vogt et al. (2019) involved educational games presented on a tablet, for which the robot provided instructions, feedback, and support, but in which—as the authors acknowledge—the robot appeared to be non-critical for the learning interaction. The robot's social behavior may matter more for conversation and storytelling-based activities than for tablet games or simpler word learning tasks. Thus, we suspect that the robot's social capabilities (such as nonverbal immediacy) can influence children's learning—as we have seen here and in multiple other studies discussed earlier—but that the influence of social behavior is moderated by other factors, such as the extent to which the robot's sociality is necessary for the learning activity to proceed smoothly (as in the case of conversation and storytelling-based activities), and the extent to which the robot's social behavior helps build rapport.

This hypothesis is supported by Lubold and colleagues' recent work with middle school children and adults, in which a social robot with vocal entrainment contributed to increased learning on math tasks, though not increases in self-reported rapport (Lubold et al., 2016, 2018; Lubold, 2017). Because the vocal entrainment served not only to match pitch and other vocal features, but also made the robot's text-to-speech voice much more expressive, these studies could not disentangle the effects of expressivity from entrainment—however, both expressivity and entrain increase the robot's social capabilities. Our results here are similar to Lubold et al.'s, in that we also found that the robot's vocal entrainment was related to learning, but unlike Lubold's work, we also found connections between the robot's entrainment and aspects of children's relationship and rapport, including increased positive emotion and language emulation. This difference could be for numerous reasons, including the different age groups studied, the different learning matter (math vs. language), and the additional social and expressive capabilities of our robot.

Our results also extend prior work showing that children learn through storytelling with peer-like robot companions in ways that are significantly different from how children learn and engage with other technologies. We are seeing a peer learning dynamic similar to that seen in child-child interactions. Children socially model and emulate the behavior of the robots, like they do with other children. For example, children are more emotionally expressive when the robot is more expressive (Spaulding et al., 2016), show more curiosity in response to a robot's increased curiosity (Gordon et al., 2015), teach new tasks to robot peers (Park and Howard, 2015), and emulate linguistic phrases and vocabulary (Kory Westlund et al., 2017b). This study extends these previous works to explore not only *whether* children will learn with and emulate a robot peer, but the *mechanisms* by which robots can influence peer learning. Rapport and relationship appear to be two such mechanisms.

### 5.4. Limitations

This study had several limitations. First, we did not control for children's individual differences, particularly with regards to learning ability, language ability, or socio-economic status, all of which may affect individual children's social interactions and learning with the robot. Furthermore, we did not obtain an equal number of children at each age group to participate in the study. Future work should examine a more homogeneous sample as well as explore the stability of results across individual differences and across ages as children grow older.

We also lacked complete story retelling data and affect data for all children. Some children did not retell the story and in a few cases, we had issues regarding the audio quality of the recorded stories. Some children's faces were not recognized by the Affdex software, and a few videos were missing or insufficiently captured a full frontal view of the children's faces, which was necessary for affect recognition. As a result, the analyses reported are underpowered. Future work should take greater effort to obtain quality audio and video recordings for all children during the study.

As mentioned in Kory Westlund et al. (2017b), the target vocabulary words were uncommon, but some children still may have known them. In particular, older children may have been familiar with some of the words, given the correlation we observed between children's age and the number of words identified correctly. The words' uncommonness may have cued children to pay attention to them; as such, future work should consider using nonce words or include a vocabulary pretest. Including a vocabulary pretest would also help ensure that children's language abilites did not differ by condition.

The robot's automated entrainment was limited to its speaking rate and pitch, so if a child was very quiet or spoke rarely, the robot would not have been able to entrain to that child. Because volume and exuberance were teleoperated, these occurred for all children. Future work could explore ways of encouraging shy children to speak up, or explore other modalities for entrainment, such as posture, gesture, facial expressions, and word use.

It is also unclear how generalizable the results are to robots with different embodiments or morphologies. The Tega robot that we used appears much like a fluffy stuffed animal, and thus is morphology could be seen as more familiar to children than a robot such as the Aldebaran NAO, which is humanoid. Children may feel a different level of comfort or uncanniness with a humanoid robot than with the Tega robot.

Finally, this study explored only a single one-on-one interaction with the robot. As such, any overall effects could be related to the novelty of the robot. However, children had the same amount of exposure to the robot in all conditions, so novelty cannot explain the differences we observed between conditions regarding the effects of entrainment and backstory.

Because learning tends to happen over time, as does the development of relationships, future work should explore longitudinal interactions to help us better understand the relationship between learning and rapport. Furthermore, children are frequently accompanied by friends and siblings in educational contexts. We do not know how multiple encounters with the robot or how interacting in groups might affect children's development of a relationship and rapport with the robot. Exploring group interactions that include multiple children, or children in concert with parents and teachers, could help us learn how to integrate robots into broader educational contexts and connect learning with peers to learning in school and at home.

## 6. Conclusion

In this work, we explored the impact of a robot's entrainment and backstory on children's engagement, rapport, relationship, and learning during a conversation and story activity. We found that the robot's rapport- and relationship-building behaviors affected children's emulation of the robot's words in their own stories, their displays of positive emotion, and their acceptance of the robot, and their perception of the robot as a social agent. This study adds to a growing body of work suggesting that the robot's social design impacts children's behavior and learning. The robot's story, use of relationship behaviors, nonverbal immediacy and rapport behaviors, social contingency, and expressivity are all important factors in a robot's social design.

## Ethics Statement

This study was carried out in accordance with the recommendations of the MIT Committee on the Use of Humans as Experimental Subjects with written informed consent from all child subjects' parents and verbal assent from all child subjects. All child subjects' parents gave written informed consent and all child subjects gave verbal assent in accordance with the Declaration of Helsinki. The protocol was approved by the MIT Committee on the Use of Humans as Experimental Subjects.

## Author Contributions

The study was conceived and designed by JK-W and CB. Data analysis was performed by JK-W. The paper was drafted, written, revised, and approved by JK-W and CB.

### Conflict of Interest Statement

The authors declare that the research was conducted in the absence of any commercial or financial relationships that could be construed as a potential conflict of interest.

## Footnotes

^1^The children who failed to complete the study were primarily younger children (one 3-year-old, five 4-year-olds, one 5-year-old, and two six-year-olds). Most were very distracted during the session and did not want to play with the robot for the full duration of the session. One 4-year-old and the 3-year-old appeared scared of the robot and did not want to interact at all, even with parental prompting. One of the 6-year-olds had accidentally signed up for the study twice, and this was not noticed until after we began the session.
