# Supplementary material for: Exploring the Effects of a Social Robot's Speech Entrainment and Backstory on Young Children's Emotion, Rapport, Relationship, and Learning
Source: Front Robot AI. 2019 Jul 9;6:54. doi: 10.3389/frobt.2019.00054 (PMC7806080; doi:10.3389/frobt.2019.00054)
Supplement: Supplementary file 1 [file Data_Sheet_1.PDF]

Detailed Procedure for: Exploring the effects of a social robot's speech entrainment and backstory on young children's emotion, rapport, relationship, and learning

Jacqueline M. Kory-Westlund [1,*] **and Cynthia Breazeal** [1]

[1] *MIT Media Lab, Massachusetts Institute of Technology, Cambridge, MA, USA*

Correspondence*:
MIT Media Lab, Massachusetts Institute of Technology, 20 Ames St., Cambridge, MA 02139, USA
jakory@media.mit.edu

1 DETAILED PROCEDURE

This section provides additional details regarding the experimental procedure for the study presented in the paper:

Kory-Westlund, J. M., Breazeal, C. (2019). Exploring the effects of a social robot's speech entrainment and backstory on young children's emotion, rapport, relationship, and learning. *Frontiers in Robotics & AI.*

Five different experimenters (three female adults and two male adults) ran the study in pairs in a quiet room in the lab. One experimenter interacted with the child. The second experimenter was present in the room, but sat back behind a laptop and did not interact directly with the child; their role was to teleoperate the robot and manage the other equipment. For each child, the interaction with the robot lasted about 20 minutes, followed by 5-10 minutes for the posttests. The interaction script and other study materials are available for download from figshare at: `http://10.6084/m9.figshare.7175273`.

Each child was greeted by the first experimenter and led into the study area. The study setup is shown in Figure 1. Some children wished their parents to stay with them (e.g., if they were particularly shy); in these cases children's parents were instructed to watch only and let their children do as much as possible by themselves. The experimenter introduced the sleeping robot to the child: "All right, this is Tega! Tega loves looking at pictures and telling stories. You're going to get to talk to Tega and read a story together!" Then the experimenter invited the child to touch the robot's fur, suggesting that maybe the fur was a little messy, and perhaps they could comb it a little together.

If the child was in the Backstory condition, the experimenter proceeded to explain that Tega sometimes had trouble hearing: "Do you see Tega's ears? Tega's ears are hiding under all the fur, so sometimes Tega's ears don't work very well. Tega sometimes has a lot of trouble hearing. You should talk to Tega in a loud and clear voice so Tega can hear you. Try to be understanding if Tega needs to hear something again." Then, in all conditions, the experimenter invited the child to try waking up the robot by saying "Wake up!" in a loud voice.

The robot interaction had four main sections: A brief introductory conversation (providing context for sharing the backstory), a conversation about pictures (providing opportunities for speech entrainment and a helping/compliance request), a sticker task (a sharing/compliance request), a storytelling activity (providing

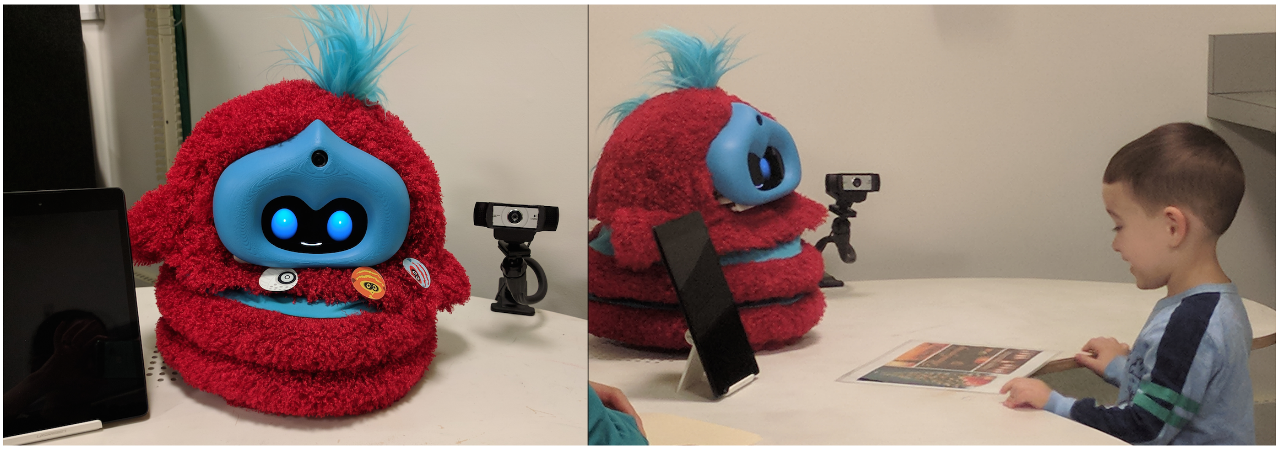

Figure 1. (A) The robot was placed on a table. The tablet was set upright to the left (when facing the robot), and the camera behind the robot and to the right. (B) A child discusses holidays with the robot in the picture conversation task. Written informed consent was obtained to use this image.

opportunities to learn words and mirror the robot's speech), and a brief closing conversation (including a goodbye gift task).

In the introductory conversation, the robot yawned, stretched, and introduced itself: "Hi, I'm Tega!" It shared personal information and prompted for disclosure in return: "My favorite color is blue. What is your favorite color?" and "Do you like to dance? I like to dance!" Following this, in the Backstory condition, the robot asked whether it could tell the child something, and said: "Sometimes I have trouble hearing and I can't always understand what people tell me. I try really hard, but sometimes I just don't hear things right. I need help and practice to get better!" Tega then asked, "Will you help me practice listening by talking and playing with me?" If the child's response was positive, the robot would say, "Oh good, thanks for understanding." If the child answered negatively, the robot asked if the child did not want to play, and if the child persisted, the experimenter would intervene and check whether the child wanted to stop the interaction entirely.

The first activity was a conversation about pictures, which was designed to provide many conversation turns for the child, and thus provide the robot with opportunities to entrain its speech to the child's. This task took approximately five minutes. It was introduced by the experimenter: "Okay [child name], you and Tega are going to look at some pictures so Tega can practice and get better at listening. You have to do at least three pictures, but you can do one more if you want." This set up a later compliance/helping task, in which the robot asked if the child would do a fourth picture with it to help it practice extra. The pictures were printed on paper and laminated; they were placed one at a time in front of the robot and child by the experimenter. The four pictures were: (1) a collage of photos of different holidays; (2) a collage of photos of children in school and reading books; (3) a picture of a playground at a park; and (4) a collage of pictures from different children's movies. For each picture, the robot introduced the picture content, e.g., "Here you can see pictures of a bunch of holidays," or "Ooh, a park!" Then the robot expressed something it liked about the picture and asked the child a question, e.g., "My favorite part of school is reading and telling stories. What's your favorite thing to do at school?" After the child responded, the robot could reply with generic listening responses, such as "Can you tell me more?", "Wow!", "Oh cool!", "Keep going," and "That sounds like so much fun!" The robot then disclosed another fact relevant to the picture and asked the child another follow-up question, e.g., "Last Christmas I got a set of Legos! What is your favorite toy?"

At two points during this activity, there were scripted moments where the robot had difficulty hearing, to reinforce the story told about it. During the first picture, the robot responded as if it had heard something the child hadn't said, saying, "Wow, I really like elephants too!" During the third picture, the robot asked, "I didn't hear that, can you say it again?"

After the third picture, the experimenter told the child, "Okay, you've finished all the pictures you had to do. Next, Tega will tell a story. But you can do another picture if you want. Do you want to look at another picture with Tega and help Tega practice?" If the child agreed, the experimenter placed the fourth picture on the table and the robot led a conversation about it as before. If the child declined, the experimenter moved on to the sticker task.

The sticker task was used to see how likely the child was to agree to a request by the robot to share a favorite object. In the sticker task, the experimenter said, "Now that you two have finished looking at pictures, you can each have a sticker. [Child name], why don't you pick your favorite sticker first?" The experimenter held out a handful of colorful stickers, with only one of each color. The child picked a sticker; the robot then said, "Hey, I want that sticker!" The experimenter responded with, "Sorry, [child name] took the only one." The robot replied, "Aww. . . Can I have your sticker?" The child could spontaneously speak or give their sticker to the robot, or decline. If the child gave their sticker, the experimenter would conveniently find a duplicate sticker in their pocket to replace it, so that the child would not have to forgo their favorite sticker. If the child declined, the robot would happily say, "Aww, that's okay, I'll just pick another one!"

Next was the storytelling activity. This task was modeled after the story retelling task used in (Kory Westlund et al., 2017): The robot asked the child if they wanted to hear a story. The robot told a story, which consisted of a 22-page subset of the wordless picture book "Frog, Where Are you?" by Mercer Mayer. The pages of the book were shown one at a time on the tablet screen. On each page, the robot said 1–2 sentences of the story. Every few pages, the robot asked a dialogic reading comprehension question about the events in the story, e.g., "Where is the deer taking the boy?", 'and "How do you think the boy feels now?" (3 questions total, decreased from the 11 questions in the prior study to decrease the length of the story activity). As in the prior study, the robot responded to children's answers with encouraging, non-committal phrases such as "Mmhm", "Good thought" and "You may be right".

We embedded six target vocabulary words (all nouns) into the story. As in the prior study, we did not test children on their knowledge of these words prior to the storytelling activity because we did not want to prime children to pay attention to these words, since that could bias our results regarding whether or not children would learn or use the words after hearing them in the context of the robot's story. We used the six key nouns identified in the original story in (Kory Westlund et al., 2017), which were replaced with the target words "gopher"(original word: animal), "crag" (rock),"lilypad" (log), "hollow" (hole), "antlers" (deer), and "cliff" (hill). Finally, like in the prior study, during assessments, we examined both children's receptive knowledge of the words as well as children's expressive or productive abilities, since children who can recognize a word may or may not be able to produce it themselves.

After the robot told the story, it asked the child if they liked the story, and then said, "I'm worried that I didn't tell the story right. I think I may have forgotten something, but I'm not sure. Do you think you could retell the story back to me so I can make sure I remembered it right?" This prompt presented an opportunity for children to retell the story, providing a measure of their story recall. During the story, the robot provided general prompts such as "Keep going!" and "And then what happened?" Twice during the retell, the robot had difficulty hearing and asked, "What? Can you say that again?" or "I didn't hear that.

Can you say it again?" Children could use the tablet while retelling the story to go through the story pages, so they could see the pictures to help them remember the story.

The final task, included during the closing conversation, was a goodbye gift. When the child had completed their retell, the robot thanked them for playing. The experimenter brought out a tray with several objects on it: a small toy frog (because the frog was present in the robot's story), a small book (because the robot expressed great interest in stories), a sticker of the robot's favorite color (blue), and an orange sticker. The experimenter said, "Okay, now you get to pick out a goodbye gift to give to Tega. Here are the different things we have. Which one do you want to give to Tega?" After the child picked an object to give to the robot, the experimenter followed up by asking why the child had picked that gift.

This completed the robot interaction. The experimenter then administered a PPVT-style vocabulary test of the six target words in the story. For each word, four pictures taken from the story's illustrations were shown to the child. The child was asked to point to the picture matching the target word. This was followed by the Inclusion of Other in Self task, adapted for children as described in (Kory-Westlund et al., 2018). In this task, children are shown seven pairs of circles that proceed from not overlapping at all to overlapping almost entirely. They are asked to point to the circles showing how close they feel to five different entities: their best friend, their parent, a bad guy they saw in a movie, their pet (or if they have no pet, their favorite toy), and the robot. Then the experimenter asked several questions taken from the Social Acceptance Scale for Kindergarten Children (Favazza and Odom, 1996; Favazza et al., 2000) regarding how accepting children might be of the robot and its hearing difficulties, as well as of other children who might have hearing difficulties. Finally, children performed a Picture Sorting Task, in which they were asked to arrange a set of eight entities along a line. The entities included a baby, a frog, a cat, a teddy bear, a computer, a mechanical robot arm, a robot from a movie (e.g., Baymax, WALL-e, or R2D2, depending on which the child was familiar with), and Tega. The line was anchored at one end with a picture of an adult human female and at the other with a picture of a table. We wanted to see where children placed the robot in relation to the other entities.

REFERENCES

Favazza, P. C. and Odom, S. L. (1996). Use of the Acceptance Scale to Measure Attitudes of Kindergarten-Age Children. *Journal of Early Intervention* 20, 232–248. doi:10.1177/105381519602000307

Favazza, P. C., 1954, Phillipsen, L., and Kumar, P. (2000). Measuring and promoting acceptance of young children with disabilities. *Exceptional Children* 66, 491–508

Kory Westlund, J. M., Jeong, S., Park, H. W., Ronfard, S., Adhikari, A., Harris, P. L., et al. (2017). Flat versus expressive storytelling: Young children's learning and retention of a social robot's narrative. *Frontiers in Human Neuroscience* 11. doi:10.3389/fnhum.2017.00295

Kory-Westlund, J. M., Park, H. W., Williams, R., and Breazeal, C. (2018). Measuring young children's long-term relationships with social robots. In *Proceedings of the 17th ACM Conference on Interaction Design and Children* (ACM), 207–218
