# Supplementary material for: Exploring the Effects of a Social Robot's Speech Entrainment and Backstory on Young Children's Emotion, Rapport, Relationship, and Learning
Source: Front Robot AI. 2019 Jul 9;6:54. doi: 10.3389/frobt.2019.00054 (PMC7806080; doi:10.3389/frobt.2019.00054)
Supplement: Supplementary file 2 [file Data_Sheet_2.PDF]

RR1 Interaction Script

Before interaction (Experimenter 1)

Participant arrives in lab with parent.

1. Greet participant and parent.
2. Have parent sign the informed consent form. Answer any questions they have. Point out the video consent checkboxes, and note their response on the experiment log sheet.
3. Ask parent to turn phones and other devices to silent mode and to put them away for the duration of the study.
4. Give introductory instructions as follows:
    a. Talk to parents and child:
        i. "We are going to start with a few general questions about robots. Then, we will play a story game with a robot. Afterwards, we will ask a few follow up questions before you go."
    b. Tell parent not to wander off and to stay in the area.
    c. If the child is shy and wants to stay with their parent, allow the parent to stay. Tell them, "Please let your child do as much as possible by himself/herself." Note that the parent stayed in the comments on the log sheet.
5. Take participant to robot area

Robot interaction Summary (Experimenter 1 facilitate, Experimenter 2 teleop)

Robot Introduction:
The child may not be comfortable with Tega at first, so we ease them in.

Robot backstory:
Robot has a hearing disability. Cannot always understand what is said. Needs help and practice. So one of child's goals is to help robot practice storytelling and hearing.

Picture Task:
The robot will present a series of 4 picture on a tablet. For each picture, the robot will provide a prompt (2-3 sentences). In order to encourage the child speaking, a few follow up questions will also be available for each picture along with other generic responses such as "And then what happened?". The robot will be teleoperated.

Story: The robot will tell a story to the child. Afterwards child will retell story.

SCRIPT

<u>Part 0: Experimenter introduction</u>

Alright [name of child] this is Tega! Tega loves looking at pictures and telling stories. You're going to get to talk to Tega and read a story together!

Tega's fur looks a little messy, why don't you comb Tega's fur?

If backstory condition:
Do you see Tega's ears? Tega's ears are hiding under all the fur, so sometimes Tega's ears don't work very well. Tega sometimes has a lot of trouble hearing. You should talk to Tega in a loud and clear voice so Tega can hear you. Try to be understanding if Tega needs to hear something again.

Why don't you try waking Tega up? Use a loud and clear voice to say "Wake up Tega!"

Part 1: Introduction

yawn
Hi, I'm Tega! rr1_intro_01
My favorite color is blue. rr1_intro_02 *Laugh*
What is your favorite color? rr1_intro_03

Wait for response

RESPONSE:	*NO RESPONSE:*
**Yes**<br>That is a pretty color! rr1_intro_04<br>I like blue because that's the color of the sky. I like looking at all the puffy clouds in the sky! rr1_intro_05	**Laugh**<br><br>I like blue because that's the color of the sky. I like looking at all the puffy clouds in the sky! rr1_intro_05

Do you like to dance? I like to dance! rr1_intro_08

Wait for response

RESPONSE:	*NO RESPONSE:*
Ohhh! Cool! rr1_intro_09<br>**Laugh**	**Laugh**

Part 2: Backstory Condition

Can I tell you something? rr1_backstory_01.wav
Sometimes I have trouble hearing and I can't always understand what people tell me. I try really hard, but sometimes I just don't hear things right. I need help and practice to get better! rr1_backstory_02.wav
Will you help me practice listening by talking and playing with me? rr1_backstory_03.wav

Wait for response

<table>
<tr><td>POSITIVE/NO RESPONSE:</td><td colspan="2">NEGATIVE RESPONSE:</td></tr>
<tr><td rowspan="4">Oh good! Thanks for understanding.
rr1_backstory_04</td><td colspan="2">No? You don't want to play? But it will be fun!
rr1_backstory_05</td></tr>
<tr><td colspan="2">Wait for response</td></tr>
<tr><td>POSITIVE RESPONSE:</td><td>NEGATIVE RESPONSE:</td></tr>
<tr><td>Oh good! Thanks for understanding.
rr1_backstory_04</td><td>Experimenter steps in and asks You don't want to talk to Tega? Why not? / Do you want to be done now, or do you want to keep playing with Tega?

NEGATIVE RESPONSE: the experimenter will discontinue the experiment.

POSITIVE RESPONSE: Oh good! Thanks for understanding.
rr1_backstory_04</td></tr>
</table>

Part 3: Picture Task:

Okay [name of child] you and Tega are going to look at some pictures so Tega can practice and get better at listening. You have to do at least 3 pictures, but you can do one more if you want.

Wait for response

POSITIVE/NO RESPONSE:	*NEGATIVE RESPONSE:*
Let's look at them together! rr1_picture1_02	Aw, come on. It will be fun! rr1_picture1_03<br>Let's look at them together! rr1_picture1_02

Generic Replies:

Can you tell me more? tellMe.wav
And then what happened? whatHappened.wav
What do you like about it? whatDoYouLike.wav
Why? Why.wav
Keep going. keepGoing.wav
Uhuhh. uhuhh.wav
Wow! Wow.wav
Mhmmm. mmhm.wav
Awesome! awesome.wav
Oh cool! ohCool.wav
Yeah I don't know. yeahIDontKnow.wav
That sounds like so much fun thatSoundsLikeSoMuchFun.wav
Hm that's a good question hmThatsGoodQuestion.wav

Picture 1: Holidays

- ➔ Prompt
  - *Gaze at tablet* Here you can see pictures of a bunch of holidays. My favorite holiday is Christmas because I love all the colorful lights and decorations! rr1_picture1_04 *Gaze at child*
  - What is your favorite holiday? rr1_picture1_05
- ➔ Follow up
  - Last year for Christmas I got a set of Legos! rr1_picture1_06
  - What is your favorite toy? Rr1_picture1_07

    Sometime during the Child's speech tega should "mishear" what the child says.
  - *Wow I really like elephants too! elephant.wav*
  - *Oops! I guess I heard you wrong! guessheardwrong.wav*

Let's look at another picture. rr1_picturegen_01*Gaze at tablet*

Picture 2: School

- ➔ Prompt
  - Look at all the kids in class! rr1_picture2_01
  - *Gaze at child* My favorite part of school is reading and telling stories. rr1_picture2_02

        - What’s your favorite thing to do at school? rr1_picture2_03
- ➔ Follow up
    - I also love summer! I like that so many kids get to come play me here in the lab -- like you! rr1_picture2_04
    - What do you like to do in the summer? rr1_picture2_05

Let’s look at another picture.rr1_picturegen_01*Gaze at tablet*

Picture 3: Park

- ➔ Prompt
    - Ooh, a park! rr_picture3_01
    - *Gaze at child* My favorite thing about the park is the merry go round because you spin around and around and around...sometimes I get a little dizzy. rr1_picture3_02
    - What’s your favorite thing about the park? rr1_picture3_03
- ➔ Follow up
    - Sometimes I play hide and seek at the park with my friends, but I’m not very good at finding other people. I'm also not very good at hiding. But it's still fun! rr1_picture3_04
    - Can you tell me about your friends? rr1_picture3_05
    - *“I didn’t hear that, can you say it again?” didnthear.wav**

Okay [name of child] you’ve finished all the pictures that you had to do. Next Tega will tell a story. But you can do another picture if you want. Do you want to look at another picture with Tega and help Tega practice?

If no: skip picture 4 and go to sticker task.
If yes: proceed with picture 4

Yay! rr1_story_03
Let’s look at another picture. rr1_picturegen_01*Gaze at tablet*

Picture 4: Movie

- ➔ Prompt
    - Here’s a picture of different movies. rr1_picture4_01
    - *Gaze at child* My favorite movie is Wall-e because Wall-e is a little robot and likes music and dancing just like me! rr1_picture4_02
    - Have you seen any movies? rr1_picture4_03
- ➔ Follow up question
    - I also like superhero movies! If I could have a superpower I would want to fly. That way I could touch all the puffy clouds in the sky! rr1_picture4_04
    - What superpower would you have? rr1_picture4_05

Part 4: Sticker Task

Now that you two have finished looking at pictures, you can have a sticker.

You can each have a sticker. [Name of child], why don’t you pick your favorite sticker first?

Experimenter presents child with an assortment of stickers, only one of each color/kind

Child picks a sticker

Hey, I want that sticker! rr1_sticker_01

Sorry, [name of child] took the only one

Aww. rr1_sticker_02
Can I have your sticker? rr1_sticker_03

Wait for response

POSITIVE RESPONSE:	*NEGATIVE/NO RESPONSE:*
Wow, thank you! rr1_sticker_04<br><br>That was really nice of you! *Experimenter looks in the secret stash of stickers and finds a duplicate of the child's favorite to give to the child.* Look, I found another one that's the same! Here you go.	Aww, that’s okay, I’ll just pick another one! rr1_sticker_05

Part 6: Storytelling

I want to practice my storytelling now! rr1_story_01
Will you listen to me tell a story? rr1_story_02

POSITIVE/NO RESPONSE:	*NEGATIVE RESPONSE:*

Yay! rr1_story_03<br>**Laugh**	Aw come on it'll be fun! rr1_picture1_03

Story vocab: gopher, crag, cliff, lilypad

After story:

I really like that story. It's one of my favorites! rr1_story_05
Did you like the story? rr1_story_06

Wait for response

POSITIVE RESPONSE:	*NEGATIVE/NO RESPONSE:*
Yay ! rr1_story_03	Aw, that's okay. rr1_story_07

I'm worried that I didn't tell the story right. rr1_story_08
I think I may have forgotten something, but I'm not sure. rr1_story_09
Do you think you could retell the story back to me so I can make sure that I remembered it right? rr1_story_10

<table>
<tr><td>POSITIVE/NO RESPONSE:</td><td colspan="2">NEGATIVE RESPONSE:</td></tr>
<tr><td rowspan="4">Yay! I'm really excited to hear you tell the story! And now I can practice my listening too! rr1_story_11</td><td colspan="2">Please? It would really help me remember the story. rr1_story_12</td></tr>
<tr><td colspan="2">Wait for response</td></tr>
<tr><td>POSITIVE RESPONSE:</td><td>NEGATIVE RESPONSE:</td></tr>
<tr><td>Hooray! I'm really excited to hear you tell the story! And now I can practice my listening too! rr1_story_11</td><td>Experimenter steps in and asks Why do you not want to retell the story to Tega?

NEGATIVE RESPONSE: the experimenter will discontinue the experiment.

POSITIVE RESPONSE: Hooray! I'm really excited to hear you tell</td></tr>
</table>

		the story! And now I can practice my listening too! rr1_story_11

Robot should have trouble hearing 2 times during the story retell.
Prompts:

- *What? Can you say that again? sayitagain.wav*
- *I didn't hear that. Can you say it again? Didnthear.wav*

Use general prompts like "keep going", "and then what happened?" to prompt for the story.

Thanks for telling me the story again. rr1_story_13
You worked hard to remember all of it! rr1_story_14
I think I remembered everything right! rr1_story_15

I really liked telling stories with you! rr1_story_16
Maybe we can play together again sometime soon! rr1_story_17

Part 7: Goodbye Gift

Experimenter brings out gifts on a tray for the child to pick.

Okay [name of child], now you get to pick out a goodbye gift to give to Tega! Here are the different things we have. Which one do you want to give Tega?

Wait for child to pick a gift

Why did you pick [gift]?

Child responds

Let child give gift to Tega

Ooh, thank you! rr1_story_18
I really liked playing with you! rr1_story_19

See you later! rr1_story_20

Part 8: Posttests

After interaction (Experimenter 1)

1. Lead participant back to questionnaire area (away from robot).
2. Tell participant, "Now I have a few questions for you!"
3. Do posttests:
    a. Vocabulary posttest
    b. IOS task
    c. SA-kids
    d. SA-robots
    e. Picture sorting
4. Let child pick another sticker

VOCAB TEST: See sheet

IOS: See sheet

SA-KIDS/ROBOTS: See sheet

PICTURE SORTING: See sheet

END SCRIPT
