# Supplementary material for: Exploring the Effects of a Social Robot's Speech Entrainment and Backstory on Young Children's Emotion, Rapport, Relationship, and Learning
Source: Front Robot AI. 2019 Jul 9;6:54. doi: 10.3389/frobt.2019.00054 (PMC7806080; doi:10.3389/frobt.2019.00054)
Supplement: Supplementary file 3 [file Data_Sheet_3.PDF]

Robot Picture Sorting Task

Overview
The goal of this task is to see how children construe the robot relative to other entities. They are asked to sort a set of pictures based on the person-like qualities of the entities in the pictures.

Coding
Look at the position of the robot relative to the person. We could also code children's explanations regarding why they sorted the pictures the way they did for how they explain the robot.

Instructions
Do you know this robot? *Hold up Wall-E. If yes, use Wall-E.*
If no:
Do you know this robot? *Hold up Baymax. If yes, use Baymax.*
If no:
Do you know this robot? *Hold up R2D2. If yes, use R2D2.*
If no, use Wall-E.

Here are some pictures of different things. Some of them are kind of like a person, like your mom or your teacher, and some aren't very much like a person at all.

Can you put each picture in the spot where it belongs? Put the things that are like a person over here, and the things that aren't like a person over here.

Remember, we want to know where you think each picture belongs. Whatever you think, that is the right answer.

Prompt for explanations of sorting order:

- Can you tell me why you're putting that one there?
- What makes that one like a person?

Write order of pictures on the answer sheet, 1 = animate (human), 10 = inanimate (table).

Pictures are:

Cat, Frog, Baby, Tega, Baymax / Wall-E / R2D2, Robot Arm, Teddy Bear, Computer

Answer sheet (or put in a spreadsheet):

1. HUMAN
2. ______________________________
3. ______________________________
4. ______________________________
5. ______________________________
6. ______________________________
7. ______________________________
8. ______________________________
9. ______________________________
10. TABLE
