# Supplementary material for: Exploring the Effects of a Social Robot's Speech Entrainment and Backstory on Young Children's Emotion, Rapport, Relationship, and Learning
Source: Front Robot AI. 2019 Jul 9;6:54. doi: 10.3389/frobt.2019.00054 (PMC7806080; doi:10.3389/frobt.2019.00054)
Supplement: Supplementary file 4 [file Data_Sheet_4.PDF]

RR1 Entrainment

These things will be entrained:

1. Speaking rate and pitch (automatic)
2. Volume (teleoperated)
3. Exuberance (teleoperated)

Instructions

Speaking rate and pitch

1. Run the audio entrainer.
2. Check the "Entrain?" box in the teleop interface.

Volume

1. Set the volume to ***0.7*** at the start of each participant's session using the new buttons in the teleop interface.
2. During the introduction and Picture 1, observe the child's personality:
    a. Are they shy, passive, reserved, or quiet? => ***quiet*** *kids*
    b. Are they loud, extroverted, active, smiley, or expressive? => ***loud*** *kids*
3. For **Picture 2**, adjust Tega's volume:
    a. For *quiet* kids: Turn Tega's volume down by 0.1 to ***0.6***
    b. For *loud* kids: Turn Tega's volume up by 0.1 to ***0.8***
4. For **Picture 3**, adjust Tega's volume again:
    a. For *quiet* kids: Turn Tega's volume down by 0.1 to ***0.5***
    b. For *loud* kids: Turn Tega's volume up by 0.1 to ***0.9***
5. Leave Tega's volume at the adjusted level for the remainder of the interaction.

Exuberance

1. During the introduction and Pictures 1, observe the child's personality:
    a. Are they shy, passive, reserved, or quiet? => ***quiet*** *kids*
    b. Are they loud, extroverted, active, smiley, or expressive? => ***loud*** *kids*
2. Then, using the animation buttons in the teleop interface, play extra animations on the robot according to the child. Pick a time to play the animation in the specified interaction phase that makes sense.
3. During **Picture 2:**
    a. For *quiet* kids: PERKUP
    b. For *loud* kids: INTERESTED
4. During **Picture 3:**
    a. For *quiet* kids: SHIMMY

    b. For *loud* kids: EXCITED
5. During **Picture 4:**
    a. For *quiet* kids: PERKUP
    b. For *loud* kids: INTERESTED
6. After the last sticker line ("Wow thank you!" or "Aww that's okay I'll just pick another one"):
    a. For *quiet* kids: SHIMMY
    b. For *loud* kids: HAPPY_DANCE
7. After the story after "It's one of my favorites!":
    a. For *quiet* kids: PERKUP
    b. For *loud* kids: HAPPY_DANCE
8. At the end after saying "See you later!":
    a. For *quiet* kids: SHIMMY
    b. For *loud* kids: HAPPY_WIGGLE
