# Supplementary material for: Exploring the Effects of a Social Robot's Speech Entrainment and Backstory on Young Children's Emotion, Rapport, Relationship, and Learning
Source: Front Robot AI. 2019 Jul 9;6:54. doi: 10.3389/frobt.2019.00054 (PMC7806080; doi:10.3389/frobt.2019.00054)
Supplement: Supplementary file 5 [file Data_Sheet_5.PDF]

Social Acceptance scale for kindergarten

adapted from Favazza 1996

Equipment
- Instruction sheet and list of questions
- Answer sheet with visual representations of "yes", "no", "maybe" (as shown in paper)

Instructions

I'm going to ask some questions about kids with disabilities. Kids who are handicapped. What do you know about kids who have disabilities, have special needs, or who are handicapped? // What else do you know about kids who have disabilities or who are handicapped?

Great. Now, after I read the question, can you point to the happy face for yes, the sad face for no, or the maybe man for maybe.

We want to know how you feel or think. Whatever you think or however you feel, that is the right answer.

If children ask questions: Let me read it again. Think about it. Then, answer it as best as you can.

Practice items (1-2)
Do you like pizza?
Do you think it will rain tomorrow?

SA - kids - pretest
1. Would you like to be good friends with a kid who can't see?
2. Would you like to be good friends with a kid who can't hear well?
3. Would you play with a kid even if they were handicapped or disabled?
4. Would you like to be good friends with a handicapped or disabled kid?

SA - robots - posttest
1. Would you like to be good friends with a robot who can't see?
2. Would you like to be good friends with a robot who can't hear well?
3. Would you play with a robot even if it was handicapped or disabled?
4. Would you like to be good friends with a handicapped or disabled robot?
